# Genome-Wide Identification of AP2/ERF Transcription Factor Family and Functional Analysis of *DcAP2/ERF#96* Associated with Abiotic Stress in *Dendrobium catenatum*

**DOI:** 10.3390/ijms232113603

**Published:** 2022-11-06

**Authors:** Yuliang Han, Maohong Cai, Siqi Zhang, Jiawen Chai, Mingzhe Sun, Yingwei Wang, Qinyu Xie, Youheng Chen, Huizhong Wang, Tao Chen

**Affiliations:** Zhejiang Provincial Key Laboratory for Genetic Improvement and Quality Control of Medicinal Plants, College of Life and Environmental Science, Hangzhou Normal University, Hangzhou 311121, China

**Keywords:** AP2/ERF, genome-wide analysis, drought stress, *Dendrobium catenatum*

## Abstract

*APETALA2/Ethylene Responsive Factor* (*AP2/ERF*) family plays important roles in reproductive development, stress responses and hormone responses in plants. However, *AP2/ERF* family has not been systematically studied in *Dendrobium catenatum*. In this study, 120 *AP2/ERF* family members were identified for the first time in *D. catenatum*, which were divided into four groups (*AP2*, *RAV*, *ERF* and *DREB* subfamily) according to phylogenetic analysis. Gene structures and conserved motif analysis showed that each *DcAP2/ERF* family gene contained at least one AP2 domain, and the distribution of motifs varied among subfamilies. Cis-element analysis indicated that *DcAP2/ERF* genes contained abundant cis-elements related to hormone signaling and stress response. To further identify potential genes involved in drought stress, 12 genes were selected to detect their expression under drought treatment through qRT-PCR analysis and *DcAP2/ERF#96*, a nuclear localized ethylene-responsive transcription factor, showed a strong response to PEG treatment. Overexpression of *DcAP2/ERF#96* in *Arabidopsis* showed sensitivity to ABA. Molecular, biochemical and genetic assays indicated that *DcAP2ERF#96* interacts with DREB2A and directly inhibits the expression of P5CS1 in response to the ABA signal. Taken together, our study provided a molecular basis for the intensive study of *DcAP2/ERF* genes and revealed the biological function of *DcAP2ERF#96* involved in the ABA signal.

## 1. Introduction

Transcription factors (TFs) are key regulators for plants to respond to various environmental stimuli and transmit stress signals. Many kinds of transcription factors, such as APETALA2/Ethylene Responsive Factor (*AP2/ERF*) [1], bHLH [2], MYB [3], Dof [4] and WRKY [5], have been proven to play vital roles in biotic and abiotic stress responses [6]. *AP2/ERF* widely exists in the plant kingdom and is one of the largest transcription factor families in plants [7], which was first discovered in *Arabidopsis* and soon found similar transcription factors in tobacco [8]. As the most representative domain of *AP2/ERF* family proteins, the AP2 domain consists of one α-helix and three β-sheets, with a range of 60–70 amino acid residues [9]. The AP2 domain can bind to some special cis-acting elements, such as dehydration responsive elements (DRE), C-repeat element (CRT) and GCC box, to regulate downstream gene expression [7,9,10]. This family is mainly divided into four subfamilies: *AP2* (APETALA2), *DREB* (Dehydration Responsive Element-Binding), *ERF* (Ethylene Responsive Element Binding protein) and *RAV* (Related to ABI3/VP) [11]. The *AP2* subfamily contains two tandem AP2 domains, which have been shown to be widely involved in floral organ development and regulation [12]. The *RAV* subfamily contains an AP2 domain and a B3 domain, which are thought to be involved in biotic and abiotic stresses, as well as its known involvement in flowering regulation [13]. The *DREB* and *ERF* subfamily both contain only one AP2 domain, but the 14th and 19th amino acids of their AP2 domains are different, resulting in a distinct affinity for different DNAs. *DREB* family proteins can specifically bind to DRE/CRT cis-acting elements, thereby regulating ABA, drought and low temperature responses, while the *ERF* subfamily proteins prefer to bind GCC-box related to ethylene response, disease resistance and abiotic stress [8].

Members of the *AP2/ERF* family have been identified and scaled in many species, including 146 in *Arabidopsis thaliana* [11], 163 in *Oryza sativa* [11], 134 in *Fagopyum Tataricum* [14], 364 in *Salix matsudana* [15], 163 in *Zingiber officinale* [16], 322 in *Triticum aestivum* [17], 189 in *Panax ginseng* [18] and 531 in *Brassica campestris* [19]. *AP2/ERF* transcription factor family has been confirmed to be involved in a variety of abiotic stress responses; for example, *AtCBF1/2/3* control low temperature responses in *Arabidopsis* [20,21], *ZmDREB2a* participates in heat response in maize [22], and *GmERF4/7* mediate salt stress response in soybean [23]. Overexpression of *OsERF71* in rice has also been found to change the root structure of plants and enhance tolerance to drought stress [24]. However, to date, no studies have clarified the role of *AP2/ERF* family genes in the response to environmental stress of *D. catenatum*, so it is very important to identify *DcAP2/ERF* by genome-wide identification approach.

*D. catenatum*, as a typical orchid plant, is highly valued because its stem contains many medicinal ingredients [25]. In addition, due to harsh growth circumstances, often attached to rocks, tree trunks and cliffs (Figure S1), *D. catenatum* has strong resistance to various stresses [26]. Therefore, studying how *D. catenatum* resists and resolves stresses from nature may provide new inspiration for studying the mechanism of plants resisting stress. To date, only a few gene families have been identified in *D. catenatum,* which are involved in stress response, including MYB [15] and GRAS [27]. With the determination of the whole genome sequences of *D. catenatum* in recent years [28,29,30], it has become possible to systematically analyze the characteristics and functions of *AP2/ERF* family in *D. catenatum*.

In this study, we first performed genome-wide identification of *DcAP2/ERF* gene family using HMMER3.0 and a total of 120 *DcAP2/ERF* genes were identified. Second, all 120 genes were comprehensively analyzed by phylogenetic analysis, gene structure and domain visualization, and promoter motif prediction. Third, we presented the expression patterns of the *AP2/ERF* family in 9 different tissues and under low temperature stress. Finally, we detected the expression of 12 *DcAP2/ERF* genes under drought stress by qRT-PCR analysis and focused on analyzing the characteristics and functions of *DcAP2ERF#96*. Our research found that the heterologous expression of *DcAP2ERF#96* causes at least 9 ABA downstream genes to be significantly inhibited, including *P5CS1* and *RD29A*, which are usually used by researchers to measure the severity of abiotic stress faced by plants [31,32,33]. We also found the protein DREB2A, which interacts with DcAP2ERF#96 in *D. catenatum*. Together, our study reveals the vital role of *AP2/ERF* family in *D. catenatum* and provides several key candidate genes related to abiotic stress.

## 2. Results

### 2.1. Identification of AP2/ERF Genes in D. catenatum

To identify the *DcAP2/ERF* family members, a combined analysis of genome-wide and full-length transcriptome-wide was performed using HMM software and SMART online. After removing the duplicate transcripts, a total of 120 *AP2/ERF* genes were finally screened and identified in the *D. catenatum* genome, and the basic information of these genes was analyzed and summarized in Table 1, including their protein length, MW, PI and subcellular location. Among these genes, the largest protein is encoded by *DcAP2ERF#47* with 733 aa, while the smallest protein is encoded by *DcAP2ERF#2* with 117 aa. (Table 1). The molecular weight (MW) of proteins ranges from 13.24 to 81.46 kDa and the isoelectric point (PI) varies from 4.35 (*DcAP2ERF#39*) to 10.10 (*DcAP2ERF#64*). According to the analysis of the aliphatic index and grand average of hydropathicity (GRAVY), the DcAP2/ERF family proteins are generally hydrophilic. To analyze subcellular localization, all AP2/ERF proteins were predicted using PSORT. The results showed that most DcAP2/ERF proteins (78.3%) are located in the nucleus, which is consistent with the localization characteristics of the transcription factor family. In addition, 18.3% of proteins were predicted to be located in cytoplasm, and 3.3% of proteins were likely to be located in mitochondria (Table 1).

### 2.2. Phylogenetic Analysis of DcAP2/ERF Families

**Figure 1:**
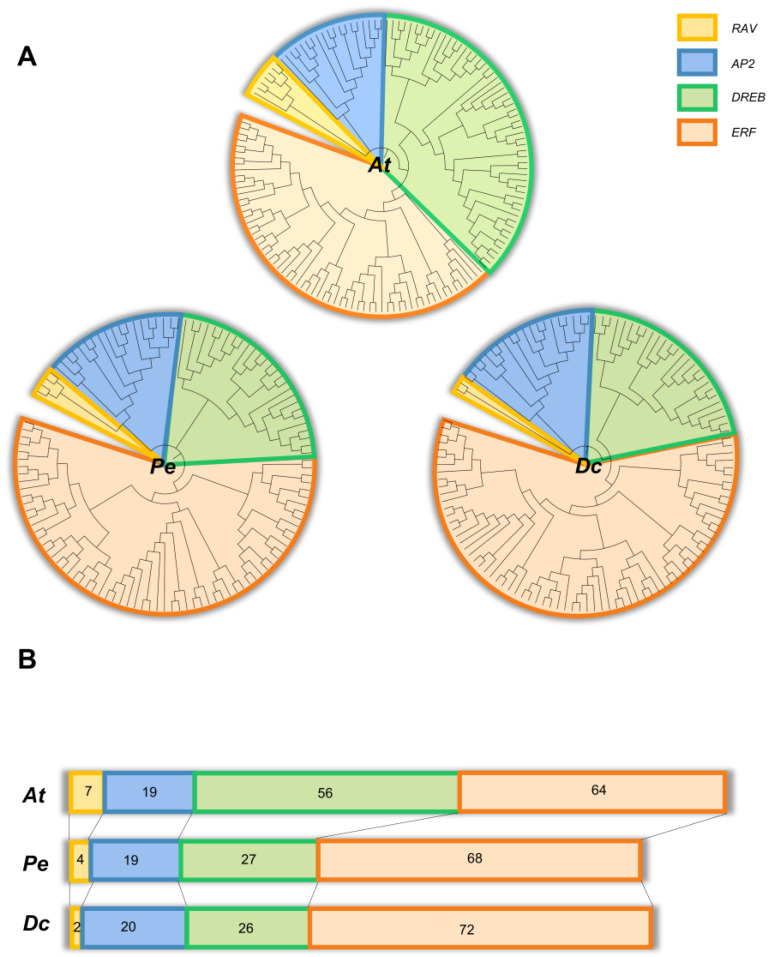
Phylogenetic analysis of AP2/ERF in *Arabidopsis thaliana*, *D. catenatum* and *Phalaenopsis equestris*. (**A**) Phylogenetic trees of three species were constructed by the neighbor-joining method with 1000 bootstrap replications. Each gene cluster was labeled with distinguishable colors. (**B**) Gene quantity and structure of different subfamilies of *AP2/ERF* family in three species.

To study the genetic phylogeny of *AP2/ERF* family in Orchidaceae, phylogenetic trees were constructed using sequences from *Arabidopsis thaliana* (146 genes), *D. catenatum* (120 genes) and *Phalaenopsis equestris* (118 genes) (Figure 1A). As shown in Figure 1, all *AP2/ERFs* of the three species can be grouped into four subfamilies, namely, *AP2*, *ERF*, *DREB* and *RAV*, which suggests that the *AP2/ERF* family is evolutionarily conserved in the plant kingdom. From the view of family size, with the deepening of evolution, the number of genes in *AP2/ERF* family has been reduced from 139 in *Arabidopsis thaliana* to 118 in *Phalaenopsis equestris* and 120 in *D. catenatum*. The size of the *RAV* and *DREB* subfamily of *D. catenatum* and *Phalaenopsis equestris* was significantly smaller than that of *Arabidopsis thaliana.* The number of *ERF* subfamily members increased slightly compared with that of *Arabidopsis thaliana*, while the gene number and tree structures of the *AP2* subfamily remained basically unchanged (Figure 1B). These changes showed that *AP2/ERF* family is conservative in the history of plant evolution, while making corresponding changes with different plant growth environments. Considering that orchids usually have stronger environmental adaptability than *Arabidopsis thaliana*, the adjustment of these gene group structures may help them cope with more complicated biotic and abiotic stresses.

### 2.3. Structure Analyses of the AP2/ERF Gene Family in D. catenatum

**Figure 2:**
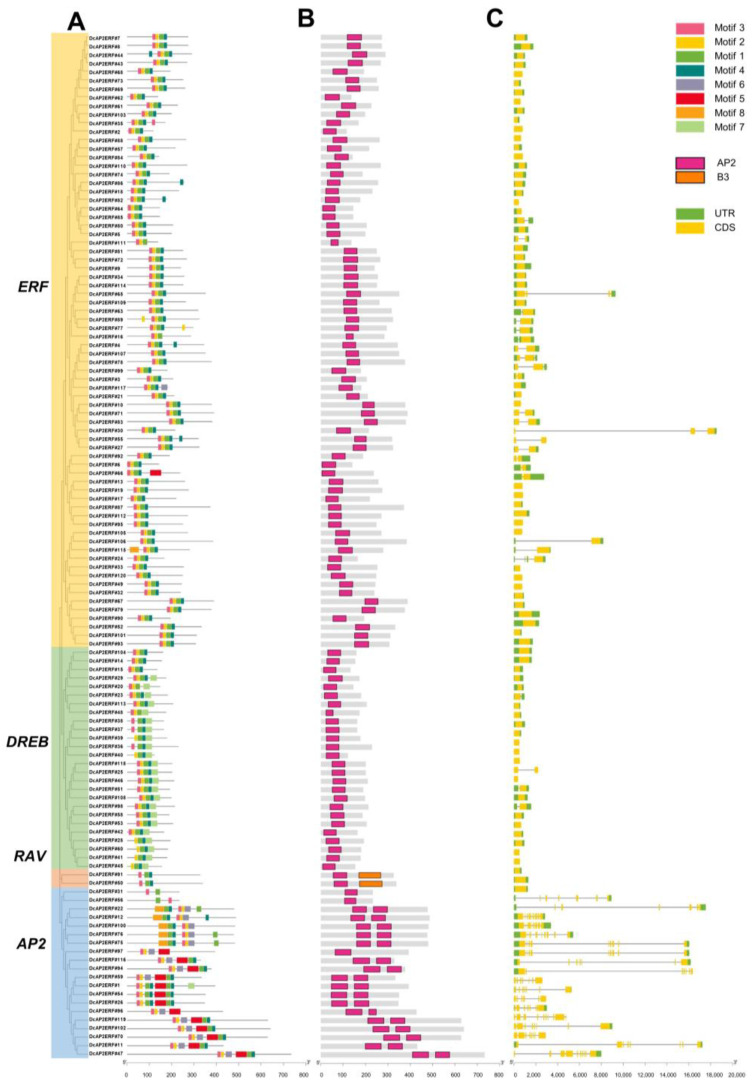
Phylogenetic relationships, conserved motifs, protein domain and gene structure in *DcAP2/ERF* genes. (**A**) Conserved motif analysis of *DcAP2/ERFs*. The four subfamilies of genes were colored on the left side, the yellow color represented the ERF subfamily, the green color represented the DREB subfamily, the pink color represented the RAV subfamily, and the blue color represented the AP2 subfamily. (**B**) Conserved domain analysis of *DcAP2/ERFs*. (**C**) Gene structure analysis of *DcAP2/ERFs*. The black lines in each gene represent the introns.

To further understand the structural characteristics of *DcAP2/ERF* family genes, the conserved motif, domain, intron and exon of *DcAP2/ERFs* were further analyzed (Figure 2). Using motif analysis, eight different motifs were predicted based on *DcAP2/ERF* protein sequences (Figure S2). We summarized the different motif patterns of each subfamily, and the results showed that motif 1, motif 3 and motif 4 exist in all four *DcAP2/ERF* subfamilies (Figure 2A). Compared with the *ERF* subfamily, the *RAV* subfamily lacks motif 2, while the *DREB* subfamily adds motif 7. Motif 7 only exists in the *DREB* subfamily and it is located outside the AP2 domain (Figure S3A). For the *AP2* subfamily containing two AP2 domains, these proteins were mainly divided into two categories. Category I is composed of motif 3, motif 2, motif 1, motif 4 (first AP2 domain) and motif 5, motif 1, motif 4 (second AP2 domain). Category II is composed of motif 8, motif 1, motif 4 (first AP2 domain) and motif 3, motif 2, motif 6 (second AP2 domain). These data suggest that the double AP2 domain of the *AP2* subfamily is not a simple duplication of the single AP2 domain, but has its own unique characteristics. For domain and structure analysis (Figure 2B,C), the characteristics of the gene members in each subfamily were consistent with previous reports [34]. It is worth noting that the intron structures of *AP2* subfamily members are very complex, which is conducive to their flexible cutting and assembly so as to cope with different situations. The diversity of the AP2 domain model may be the result of gene evolution and selection. This partly explains why different subfamilies have preferences for different cis-acting elements [8].

Combined with the secondary structure of the AP2 domain, we further analyzed the motif of a typical AP2 domain (Figure S3B). Motif 3, motif 2 and the front half of motif 1 are distributed with one β-sheet, respectively. The back half of motif 1 and the first half of motif 4 form one α-helix. These secondary structures together form a complete AP2 domain. These findings are consistent with the previous description of the AP2 domain [35]. In addition, these results showed that although *AP2/ERF* family is complex at the motif level, it is still conserved in domain composition.

### 2.4. Cis-Acting Element Analysis of DcAP2/ERF Gene Promoters

**Figure 3:**
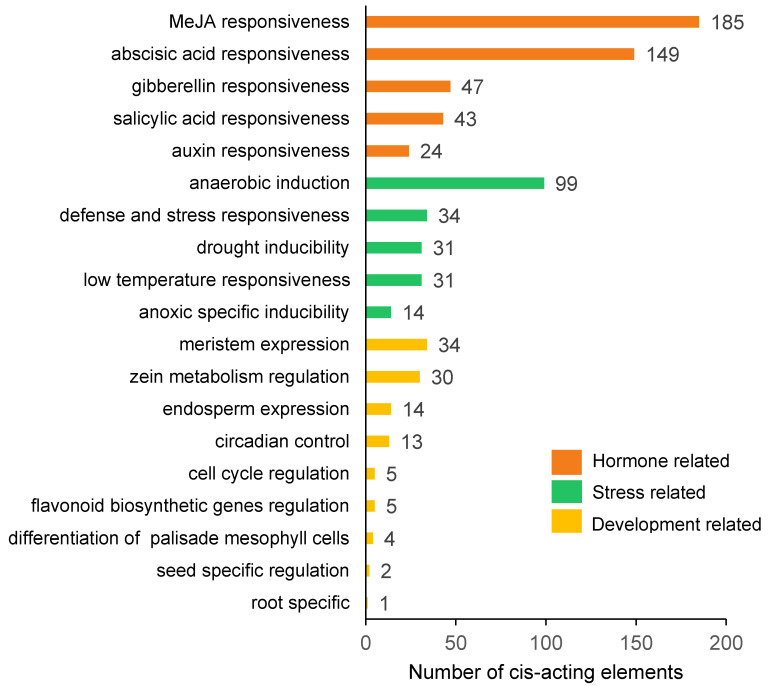
Classification and analysis of cis-acting elements in the promoter regions of *DcAP2/ERF* genes. The 2 kb region upstream of the genes was analyzed using the PlantCARE website. Graphs in different colors represent different classes of cis-acting elements. The numbers indicate the amount of each cis-acting element.

To explore the putative functions of *AP2/ERF* family genes in *D. catenatum*, the 2000-bp upstream sequences of 120 genes were extracted to analyze the potential cis-elements. The predicted TF-binding motifs were classed into three categories: hormone-related elements, stress-related elements, and growth and development-related elements (Figure 3). Hormone response, such as auxin-responsiveness, ABA-responsiveness, GA-responsiveness, MeJA-responsiveness and SA-responsiveness. From the view of hormone response-related elements, a total of 113 genes have hormone response elements in the promoter region (Table S3), indicating that *DcAP2/ERF* family genes may be widely involved in hormone response pathways. Among these elements, the number of elements responding to MeJA and ABA was enriched, reaching 185 and 149, respectively, which indicated that *DcAP2/ERF* genes may respond to biotic and abiotic stress rapidly. Stress response-related elements were associated with anaerobic induction, drought induction, low temperature responsiveness and anoxic specific induction. In addition, the promoters of 68 genes in *DcAP2/ERF* family contain growth- and development-related response elements associated with meristem expression, zein metabolism, endoperm expression, circadian control, cell cycle regulation, flavonoid biosynthetic gene regulation, and others. Meristem expression elements and zein metabolism regulation elements accounted for the largest proportion, reaching 32 and 31, respectively (Figure 3).

### 2.5. GO Annotation Analysis of DcAP2/ERF Family Proteins

To further understand the molecular function of DcAP2/ERF proteins, GO annotations were conducted in this study. According to gene ontology, genes or gene products have three main characteristics: cell composition, molecular function and biological process. Their terminology labels can help us understand the protein functions [36]. Here, a total of 75 DcAP2/ERF proteins were assigned 20 GO terms, including 4 cellular component terms, 2 molecular function terms and 14 biological process terms (Figure S4, Table S2). Under the cellular component category, 32 proteins were identified as ‘cell’ (GO: 0005623), ‘cell part’ (GO: 0044464) and ’organelle’ (GO: 0043226). Under the molecular function category, 50 and 75 proteins were annotated for ‘binding’ (GO: 0005488) and ‘transcription regulator activity’ (GO:0140110), respectively. Under the biological process category, all annotated proteins are involved in ‘metabolic processes’ (GO: 0008152), ‘cellular processes’ (GO: 0009987) and ‘biological regulatory’ (GO: 0065007). In addition, there are 53 proteins involved in ‘response to stimulus’ (GO: 0050896). In summary, DcAP2/ERF proteins are a typical family of transcription factors with the potential ability to bind downstream DNA and regulate various physiological processes.

### 2.6. Expression Patterns of DcAP2/ERF Genes under Different Stresses

**Figure 4:**
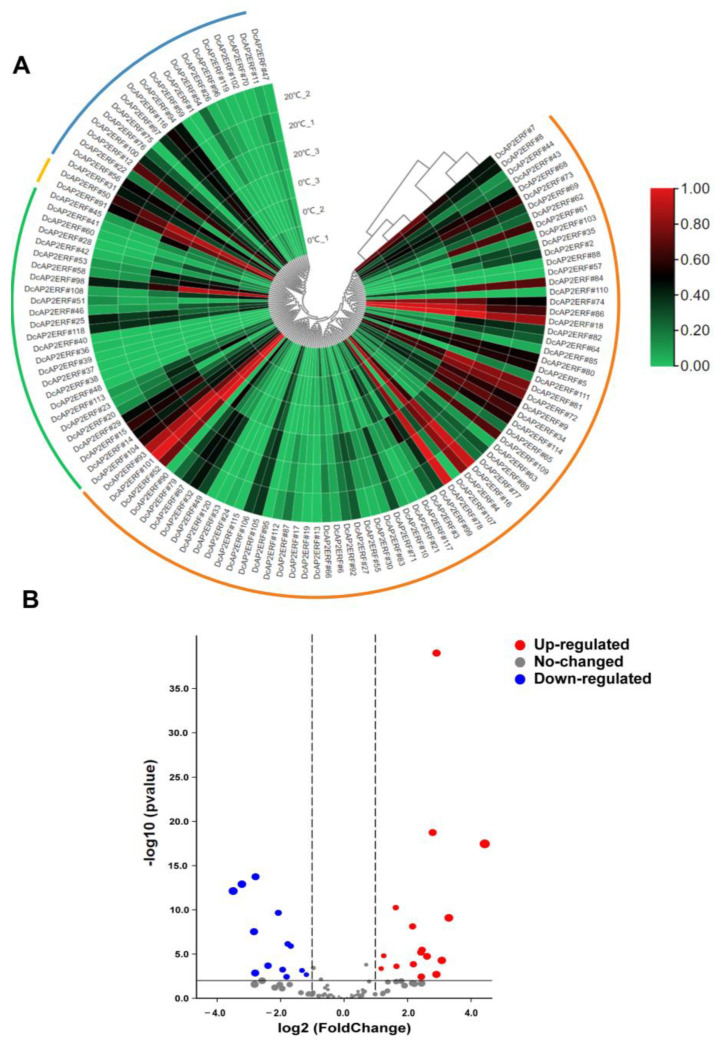
Expression of *DcAP2/ERF* genes in response to cold stress. (**A**) Heat map showing the expression pattern *DcAP2/ERF* genes in leaves under cold stress for 20 h. The red, green, yellow and blue arcs in the outer ring of the heat map represent the range of *ERF*, *DREB*, *RAV* and *AP2* subfamily, respectively. (**B**) The volcano plot showing the upregulation and downregulation of genes under low-temperature treatment.

To study the expression pattern of *DcAP2/ERF* family genes in response to hormone and environmental stresses, the transcriptomic data of *D. catenatum* under MeJA and low-temperature treatments were analyzed. In chilling stress, a total of 29 genes showed significant changes under chilling treatment, of which 16 genes were significantly upregulated, especially *DcAP2ERF#52*, *DcAP2ERF#7* and *DcAP2ERF#98* (Table S4), and 13 genes were downregulated (Figure 4B), especially *DcAP2ERF#84, DcAP2ERF#109* and *DcAP2ERF#61*. These data suggest that these genes may play important roles in the cold stress response and can be considered candidate genes for further study of cold stress biology.

**Figure 5:**
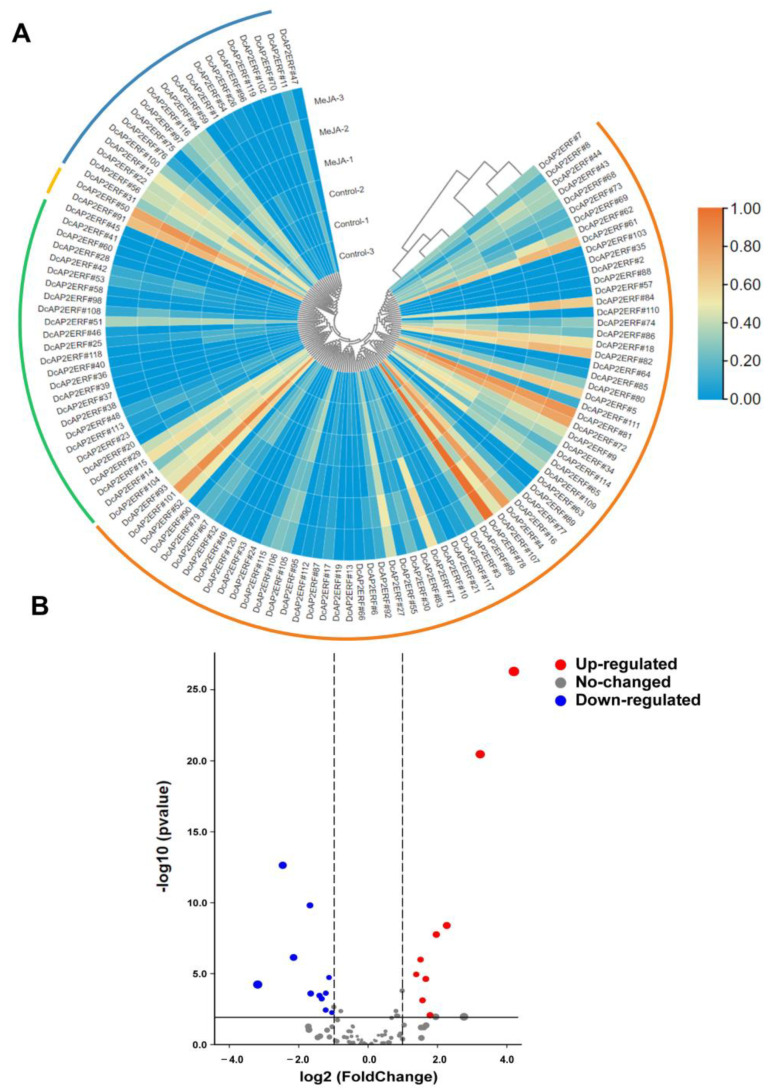
Expression of *DcAP2/ERF* genes in response to MeJA. (**A**) Heat map showing expression pattern *DcAP2/ERF* genes under 1 mM MeJA. The red, green, yellow and blue arcs in the outer ring of the heat map represent the range of *ERF*, *DREB*, *RAV* and *AP2* subfamily, respectively. (**B**) The volcano plot showing the upregulation and downregulation of genes under 1 mM MeJA treatment.

For the MeJA treatment, the repetitions of three experimental groups and three control groups were clustered into two groups (Figure 5A). Under MeJA treatment, 9 genes were upregulated, and 11 genes were downregulated (Figure 5B). Among these genes, the expression of *DcAP2ERF#44* and *DcAP2ERF#71* was highly increased (about 18 times and 9 times, respectively) compared with the control (Table S5). This suggests that they may be strongly induced by JA signaling and perform important functions in *D. catenatum*.

### 2.7. Expression Patterns of DcAP2/ERF Genes in Different Organs

To study the tissue expression pattern and reveal the related function of *DcAP2/ERF* genes, we analyzed the transcriptome data of *DcAP2/ERFs* in different tissues (Figure S5). Among all 120 *DcAP2/ERF* genes, 14 *DcAP2/ERF* genes were constitutively expressed, 11 genes tended to be expressed in roots (including green root tip and white part of root), stem or leaves, and 34 genes tended to be expressed in flower organs (including sepal, labellum, gynostemium and pollinia). In addition, by the cluster analysis of tissues and organs, the expression pattern of flower organs was distinguished from roots, stems, leaves and their related tissues. The expression pattern of gynostemium is similar to that of sepal among these *DcAP2/ERF* genes, except *DcAP2ERF#74, DcAP2ERF#64, DcAP2ERF#55, DcAP2ERF#50, DcAP2ERF#22* and *DcAP2ERF#26.* It is worth mentioning that the expression pattern of *DcAP2/ERF* genes in pollen is obviously different from those in other tissues. Some genes, such as *DcAP2ERF#117, DcAP2ERF#24, DcAP2ERF#40, DcAP2ERF#41* and *DcAP2ERF#59,* are specifically expressed in pollen, indicating that these genes play important roles in pollen development.

### 2.8. Expression Pattern of DcAP2/ERFs under PEG6000 Treatment

**Figure 6:**
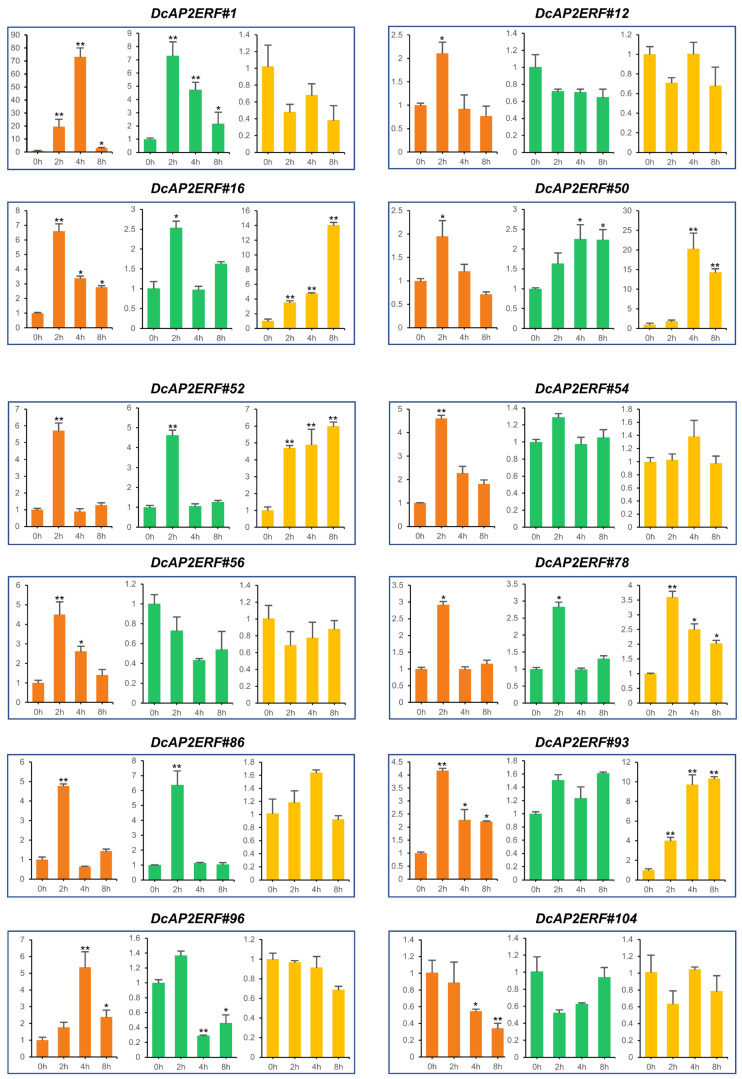
Expression patterns of 12 selected genes in leaves, stems and roots under 20% PEG6000 treatment. The orange color represented leaves, the green color represented stems, and the yellow color represented roots. *DcACTIN* was used as an internal control. Values are presented as means ± SD (*n* = 3). (* *p* < 0.05, ** *p* < 0.01, Student’s *t*-test).

Many ABA response elements in *DcAP2/ERF* family gene promoters and the excellent drought resistance of *D. catenatum* allow us to suspect that some *DcAP2/ERF* family genes are involved in the drought-tolerant response. Based on the expression of these genes in different transcriptomes (Figure 4, Figure 5 and Figure S5), we finally selected 12 genes of interest to test their expression levels under drought stress (Figure 6). After 20% PEG6000 treatment for 0 h, 2 h, 4 h and 8 h, respectively, leaves, stems and roots of *D. catenatum* were collected for RNA extraction and qRT-PCR analysis. As shown in Figure 6, most of the genes responding to drought are found in leaves, with 11 genes, while only 7 and 5 genes showed increased expression in stems and roots, respectively, under drought stress. Among these genes, four genes (*DcAP2ERF#16*, *DcAP2ERF#50*, *DcAP2ERF#52*, *DcAP2ERF#78*) showed obvious increased expression in all three tissues under drought stress. Interestingly, *DcAP2ERF#1* and *DcAP2ERF#86* only increased in leaves and stems, while they had no changes in roots, indicating that these genes may play different roles in *D. catenatum* in response to drought stresses. In addition, according to the differences in the expression levels and response times of these genes in the three tissues, we can infer that the three kinds of tissues showed different sensitivities in the face of drought stress.

### 2.9. DcAP2ERF#96 Encodes a Conserved AP2 Domain Transcription Factor Localized in the Nucleus

**Figure 7:**
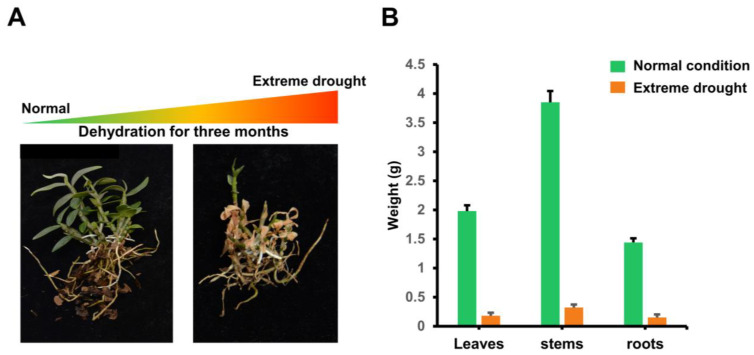
The growth status of *D. catenatum* stems is strongly affected by drought stress. (**A**) The growth status of *D. catenatum* under normal and extreme drought conditions. (**B**) Statistics on biomass loss under different degrees of drought stress.

**Figure 8:**
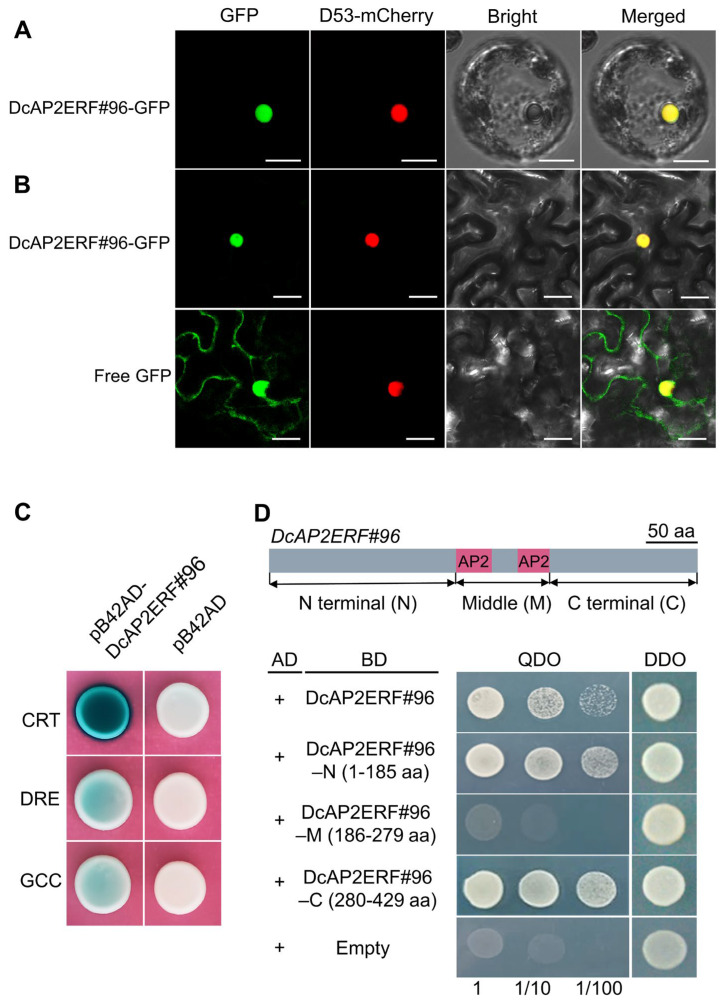
*DcAP2ERF#96* exhibits significant transcription factor characteristics. (**A**,**B**) Subcellular localization of DcAP2ERF#96 protein in *D. catenatum* protoplasts (**A**) and in the leaf epidermal cells of *N. benthamiana* (**B**). D53-mCherry was used as a nuclear marker. Bars = 20 μm. The fluorescence signals were detected by confocal microscopy. (**C**) Yeast one-hybrid verification of the binding of DcAP2ERF#96 protein to three tandem repeats of CRT box, DRE box and GCC box. (**D**) Yeast two-hybrid validation of DcAP2ERF#96 protein self-activation regions. The transformed yeast cells were plated on DDO (SD/-Trp/-Leu) and QDO (SD/-Trp/-Leu/-His/-Ade).

To further confirm the related genes involved in drought stress in the *AP2/ERF* family, we measured the biomass differences in several tissues of *D. catenatum* under drought stress and normal conditions (Figure 7). The results showed that the growth of the stem was significantly inhibited under three-month drought conditions (Figure 7A). In addition, stems, as the main medicinal parts of *D. catenatum,* had the most obvious changes in biomass under drought stress (Figure 7B). Based on this finding, we selected *DcAP2ERF#96* as a potential gene for further study, which showed specifically decreased expression in the stem under PEG6000 treatment, as shown in Figure 6. Sequence analysis revealed that *DcAP2ERF#96* is an AP2-like ethylene-responsive transcription factor. Subcellular localization in *D. catenatum* protoplasts and tobacco leaf cells showed that the DcAP2ERF#96 protein was located to the nucleus (Figure 8A,B). Previous studies have shown that *AP2/ERF* transcription factors can bind to GCC (AGCCGCC), DRE (GCCGAC), and CRT (ACCGAC) boxes to regulate downstream gene expression [6]. To confirm this, a Y1H assay was performed, and the results showed that DcA*P2ERF#96* has a strong ability to bind to the CRT box. In addition, it also has weak binding activity to DRE and GCC box (Figure 8C), indicating that the binding motifs of *DcAP2ERF#96* were conserved in *D. catenatum*. A transactivation activity assay showed that DcAP2ERF#96 had self-activating properties, and the self-activating regions existed in the N terminal (1–185 aa) and C-terminal (280–429 aa) of DcAP2ERF#96, while not the middle region (186–279 aa), which contain two AP2 domains (Figure 8D). These results confirm that DcAP2ERF#96 is a nuclear-localized transcription factor.

### 2.10. DcAP2ERF#96 Interacted with DcDREB2A and Negatively Regulate ABA Signaling in Arabidopsis

**Figure 9:**
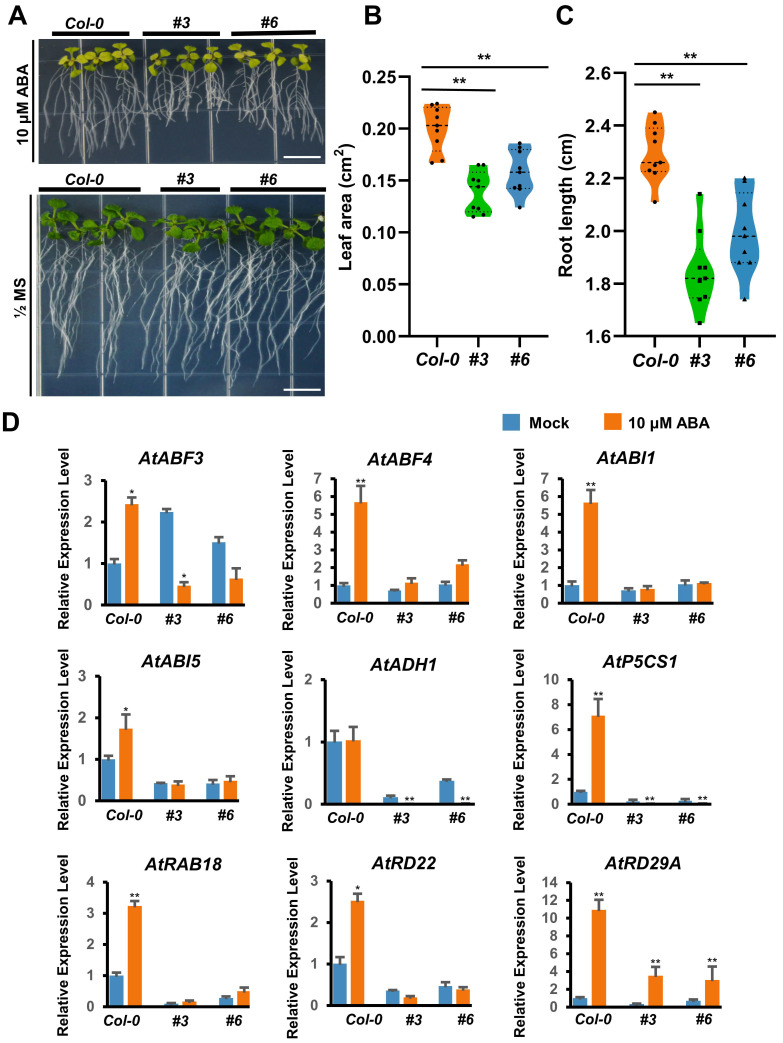
*DcAP2ERF#96* overexpression lines showed sensitivity to ABA. (**A**) 10-d-old seedlings were transferred to vertical plates containing ^1^/_2_ MS + 10 μM ABA and its control (^1^/_2_ MS). Phenotypes were observed and recorded one week later. Bars = 1 cm. (**B**,**C**) Leaf area (**B**) and root length (**C**) statistics of *Col-0* and *DcAP2ERF#96* overexpression lines under 10 μM ABA treatment (*n* = 9). (**D**) Relative expression levels of ABA signaling pathway-related genes in *Col-0* and overexpression lines after 10 μM ABA treatment for one week. *AtACTIN2* was used as an internal control. Values are presented as means ± SD (*n* = 3). * *p* < 0.05, ** *p* < 0.01, Student’s *t*-test.

**Figure 10:**
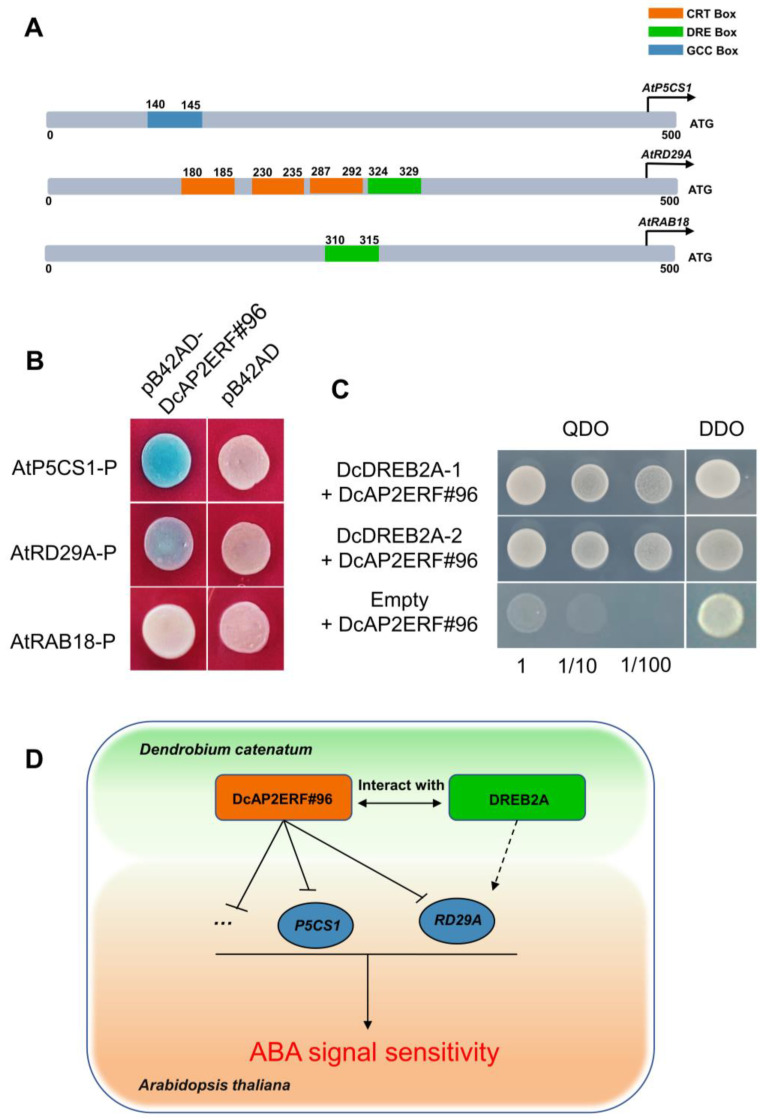
*DcAP2ERF#96* interacts with *DcDREB2A* and inhibits *P5CS1* and *RD29A* in *Arabidopsis thaliana*. (**A**) Distribution of DRE, CRT and GCC motifs in ABA-related gene *P5CS1*, *RD29A* and *RAB18* promoters. (**B**) Yeast one hybrid verification of the binding of *DcAP2ERF#96* to *P5CS1*, *RD29A* and *RAB18* promoters. The pB42AD-DcAP2ERF#96 and pLacZi2μ with different promoters were co-transformed into the EGY48 yeast strain. The transformed yeast cells were plated on DDO (SD/-Ura/-Trp) with X-Gal. (**C**) Yeast two hybrids verified the interaction of DcAP2ERF#96 with DREB2A-1 and DREB2A-2. The transformed yeast cells were plated on DDO (SD/-Trp/-Leu) and QDO (SD/-Trp/-Leu/-His/-Ade). (**D**) Regulatory model for the regulation of the ABA signal by *DcAP2ERF#96* in *Arabidopsis thaliana* and *D. catenatum*. Overexpression of *DcAP2ERF#96* inhibits the expression of ABA downstream genes such as *RD29A* and *P5CS1* by binding to their promoters, thereby affecting plant sensitivity to ABA signaling. DcAP2ERF#96 can interact with DREB2A protein in *D. catenatum*, whose homologous gene in *Arabidopsis* has been reported to positively regulate the expression of *RD29A*.

To further investigate the function of *DcAP2ERF#96* in response to drought, the two overexpression lines of *Arabidopsis thaliana* #3 and #6 with the highest gene expression levels were selected for further study. Compared with wild-type (*Col-0*), the leaf area and root length of the two overexpression lines were significantly reduced in 1/2 MS with 10 μM ABA, suggesting that *DcAP2ERF#96* had an ABA-sensitive phenotype (Figure 9A–C). To further explore whether *DcAP2ERF#96* participates in the ABA signaling pathway in response to drought, the expression of 9 genes related to the ABA signaling pathway was selected and detected under ABA treatment (Figure 9D). Results showed that all 9 genes except *AtADH1* were significantly upregulated after 10 μM ABA treatment in *Col-0*. However, the relative expression levels of these genes were significantly reduced in *DcAP2ERF#96* overexpression lines compared with *Col-0*, which confirmed our hypothesis that *DcAP2ERF#96* acts as a transcriptional repressor to negatively regulate ABA signaling under drought stress in plants. Cis-elements analysis of these 9 gene promoters showed that three gene promoters contain DRE, CRT and GCC box: The *AtP5CS1* promoter contains one GCC box; the *AtRD29A* promoter contains three CRT boxes and one DRE box; and the *AtRAB18* promoter contains one DRE element. All the elements are located within the range of 500-bp upstream sequences of genes. (Figure 10A) Y1H results showed that *DcAP2ERF#96* can directly bind to the promoter of *P5CS1* and *RD29A* in yeast, but there is no indication that it can combine with *RAB18* promoter (Figure 10B). At the same time, our Y2H results confirmed that DcAP2ERF#96 interacts with DREB2A-1 (LOC110112950) and DREB2A-2 (LOC110113533), which have been reported to regulate the expression of *RD29A* in *Arabidopsis* [37] (Figure 10C). These results suggested that *DcAP2ERF#96* negatively regulates the expression of *P5CS1* and *RD29A* in *Arabidopsis thaliana*, thereby affecting its response to the ABA signal (Figure 10D).

## 3. Discussion

*D. catenatum*, a precious Chinese herbal medicine of Orchidaceae, has great medicinal and economic value [27]. *AP2/ERF* family genes are widely involved in various physiological activities and biotic/abiotic stress responses of plants. However, the role of *AP2/ERF* family genes in *D. catenatum* remains uncertain. In this study, 120 *DcAP2/ERF* genes were detected, and their phylogenetic relationship, domain composition, gene structure, cis-acting elements and gene ontology were analyzed. Furthermore, the tissue expression profile and different responses of *DcAP2/ERF* family genes under cold environment, MeJA treatment and simulated drought were also explored. Based on these results, we discussed the potential functions of some genes in *D. catenatum.*

In phylogenetic analysis, we found obvious structural similarities of phylogenetic trees and adjustment of subfamily proportion among *Arabidopsis thaliana*, *D. catenatum* and *Phalaenopsis equestris* (Figure 1), which indicated that orchidaceae had a conserved evolutionary process. We also found that the number of introns in the *AP2* subfamily was quite greater than those in other subfamilies (Figure 2). Previous studies have shown that introns may be gradually lost in the process of evolution [38]. According to this theory, we speculate that the *AP2* subfamily may be one of the origins of the evolution of other subfamilies. Other studies have shown that the AP2 domain in plant genes may be derived from bacterial or viral HNH endonucleases, and various subfamilies have gradually been generated during the long evolutionary process [39]. In general, the evolutionary relationship between the various *AP2/ERF* subfamilies still remains to be explored.

Transcriptional regulation plays a leading role in the regulation of gene expression, which is mainly controlled by its cis-acting element on the gene promoter [40,41]. We analyzed the cis-acting elements in all *DcAP2/ERF* family promoter regions (Figure 3) and described their functions by gene ontology (Figure S4). The results showed that the cis-acting elements responding to MeJA and ABA accounted for the largest proportion, which was similar to the promoter characteristics of *AP2/ERF* family in other plants like wheat [17] and ginger [16]. At the same time, gene ontology also analyzed DcAP2/ERF proteins that have typical features of the general transcription factor families and have the ability to respond to stimuli and signal transduction.

Drought is a common abiotic stress that can cause serious damage to the growth and development of plants [42]. Drought stress will directly lead to cell dehydration, which produces an ABA signal and causes a series of tolerance responses in plants, including promoting stomatal closure of guard cells and regulating the expression of related genes [43]. Previous studies have suggested that some *AP2/ERF* family genes are closely related to drought stress and ABA [44,45]. We selected 12 genes of interest of *DcAP2/ERF* family, and tested their expression through PEG6000 treatment simulating an arid environment (Figure 6). The results confirmed our hypothesis that the expression of most selected genes increased under simulated drought conditions. As the most strongly upregulated gene, the homologous gene of *DcAP2ERF#1* in *Arabidopsis* has been confirmed to control the synthesis of epidermal wax [46], and the increase of epidermal wax in *D. catenatum* leaves may be beneficial in reducing water loss and surviving longer under drought stress. In the stem, the changes in the expression levels of all tested genes were significantly smaller than those in leaves and roots, which may be due to the fact that stems are rich in polysaccharides, polyphenols and other substances and have strong resistance to drought stress. After comparing the biomass of different tissues of *D. catenatum* under drought conditions, the stem is considered to play a key role in the process of *D. catenatum* resisting drought stress (Figure 7). At the same time, we noticed a decrease in the expression of *DcAP2ERF#96* in stems under simulated drought conditions (Figure 6). Molecular biology and transgenic techniques including subcellular localization, Y1H, Y2H and transgenic experiments confirmed that *DcAP2ERF#96* functions as a transcriptional repressor to regulate the ABA signaling pathway and interacts with DREB2A in *D. catenatum*. The downregulation of its expression in stems may be one of the self-rescue responses of *D. catenatum* under drought stress, which is conducive to the transmission of ABA signals in the whole plant. (Figure 8, Figure 9 and Figure 10). In addition, the expression of some genes downstream of ABA, such as *AtABI1*, *AtABI5* and *AtADH1,* were also significantly reduced (Figure 9D), which may be caused by *DcAP2ERF#96* indirect regulation, but this speculation remains to be further tested. In general, this study systematically analyzed the *DcAP2/ERF* family of *D. catenatum* and preliminarily studied the role of *DcAP2ERF#96* in the ABA signaling pathway. Further functional verification should be carried out to understand the role of *DcAP2/ERF* family genes under different conditions.

## 4. Materials and Methods

### 4.1. Identification of AP2/ERF Genes in D. Catenatum

To perform genome-wide identification of the *DcAP2/ERF* gene family, the whole *D. catenatum* genome and its annotation information were downloaded from NCBI (https://www.ncbi.nlm.nih.gov/, accessed on 1 November 2022). The Hidden Markov model (HMM) of the AP2 domain (pfam: PF00847) and B3 domain (pfam: PF02362) were downloaded from the Pfam database (http://www.sanger.ac.uk/Software/Pfam/, accessed on 1 November 2022). The potential *DcAP2/ERF* genes were searched by HMMER3.0 (e-value < e^−5^). All screened sequences were verified by the SMART database (http://smart.embl-heidelberg.de/, accessed on 1 November 2022) [47]. After removing duplicate transcripts, a total of 120 *AP2/ERF* family genes were found in *D. catenatum* and named as *DcAP2ERF#1* to *DcAP2ERF#120*. Basic information, such as the isoelectric point and molecular weight of genes, was analyzed by ExPASy (https://www.expasy.org/, accessed on 1 November 2022). The subcellular localization of genes was predicted using PSORT (https://www.genscript.com/psort.html, accessed on 1 November 2022).

Using the same method, we identified 118 *AP2/ERF* family genes from *Phalaenopsis equestris.* The 146 *AP2/ERF* genes of *Arabidopsis thaliana* were downloaded from PlantTFDB (http://planttfdb.gao-lab.org/index.php, accessed on 1 November 2022) [48].

### 4.2. Phylogenetic Tree Analysis of the DcAP2/ERF Protein

The phylogenetic tree was constructed by MEGA7 using the neighbor-joining method with a bootstrap test (1000 replicates). Multiple sequence alignments of all proteins were completed by MUSCLE before phylogenetic analysis [49]. For the graphic display, the final phylogenetic trees were modified using ITOL (https://itol.embl.de/, accessed on 1 November 2022).

### 4.3. Gene Structure, Conserved Domain, Motif and Promoter Analyses of AP2/ERF Family Members

The gene structure information was directly generated by TBtools according to the gene annotation file. The conserved domain information of *AP2/ERF* family proteins was calculated by Batch CD-Search (https://www.ncbi.nlm.nih.gov/Structure/bwrpsb/bwrpsb.cgi, accessed on 1 November 2022) [50]. The conserved motif of the full length of AP2/ERF proteins was analyzed using the online MEME website (https://meme-suite.org/meme/tools/meme, accessed on 1 November 2022) [51] by the zero or one occurrence per sequence (zoops) strategy and set the number of searchable motifs to 8. Other parameters are website default settings 4.4. Cis-acting element analyses of DcAP2/ERF family genes

The promoter sequences (2000-bp upstream of gene) were obtained from the *D. catenatum* genome. All cis-acting elements were calculated by PlantCARE (https://bioinformatics.psb.ugent.be/webtools/plantcare/html/, accessed on 1 November 2022) [52].

### 4.4. Plant Materials and Methods of Stress Treatment

‘Honggan ruanjiao’ a well-known *D. catenatum* variety, was used in this study. In order to detect the expression pattern of *DcAP2/ERF* family under drought conditions, 6-month-old seedlings were treated with 20% PEG6000 to mimic drought conditions. After treatment for 0, 2, 4 and 8 h, respectively, three samples (root, stem and leaf) were harvested and frozen in liquid nitrogen for RNA extraction.

In addition, *DcAP2ERF#96* was transformed into *Arabidopsis thaliana* to obtain overexpression lines. All overexpression lines were selected by hygromycin and the relative expression level of these lines was detected by qRT-PCR (Figure S6).

### 4.5. RNA Extraction and Quantitative/Real-Time-PCR (qRT-PCR) Analysis

The total RNA of plant materials was extracted using Trizol reagent (Coolaber, Beijing, China). One microgram of total RNA was used to synthesize cDNA with HiScript II Q Select RT SuperMix for qRT-PCR (Vazyme, Nanjing, China). qRT-PCR was performed on a CFX384 real-time system (BIO-RAD, Hercules, CA, USA) with ChamQ Universal SYBR qPCR Master Mix (Vazyme, Nanjing, China). The experiments were repeated three times independently. The *actin2* gene was used as an internal control. The primers used in this assay are listed in Table S1.

### 4.6. Expression Pattern Analysis of DcAP2/ERF Genes

To reveal the expression patterns of all *AP2/ERF* family genes in *D. catenatum*, the raw RNA-seq data were downloaded from the NCBI Sequence Read Archive (SRA) database. The transcriptome data of root (SRX2938667), stem (SRR4431600), leaf (SRR4431601), white part of root (SRR4431598), Green root tip (SRR4431599), sepal (SRR4431597), labellum (SRR4431602), pollinia (SRR5722145) and gynostemium (SRR4431596) were obtained for tissue expression analyses [29]. The raw RNA-seq data of leaves under 20 °C conditions (SRR3210630, SRR3210635 and SRR3210636) and 0 °C conditions for 20 h (SRR3210613, SRR3210621 and SRR3210626) were downloaded for low temperature stress analyses [53]. By clustering, six groups of transcriptomic data were grouped into two categories according to temperature, indicating that the experimental data were reliable and had significant differences between the control and chilling treatments (Figure 4A). The raw RNA-seq data of 1mM MeJA (SRR14635790, SRR14635791, SRR14635792) treatment for 4 h and control (SRR14635793, SRR14635796, SRR14635797) were downloaded for hormone treatment analyses [54]. All raw data were converted by the Kallisto method and normalized by transcripts per million (TPM). Differential expression analysis and volcano plot drawing were visualized using Tbtools.

### 4.7. Gene Ontology Annotation Analysis

Gene Ontology (GO) analysis of *DcAP2/ERF* family proteins was done by eggNOG-mapper (http://eggnog-mapper.embl.de/, accessed on 1 November 2022) and visualized by the WEGO website (https://wego.genomics.cn/, accessed on 1 November 2022). The GO annotation results are listed in Table S2.

### 4.8. Subcellular Localization of DcAP2ERF#96

The full-length coding sequences of *DcAP2ERF#96* were amplified and cloned into the pEarleyGate 101 vector to produce the DcAP2ERF#96-GFP fusion construct. To observe the localization in protoplasts of *D. catenatum*, young leaves of six-month-old *D. catenatum* were used for protoplast preparation and transformation. 10 μg plasmid was transformed into 100 μL protoplast suspension, and fluorescence was observed after 12 h [55]. To observe the localization in tobacco, four-week-old *N. benthamiana* was used for gene transient expression. The activated GV3101 *Agrobacterium* containing the constructed plasmid was incubated with infection solution (10 mM MES, 10 mM MgCl_2_ and 200 µM AS) for 2 h and then injected into the young leaves of tobacco (*N. benthamiana*). Fluorescence was observed at 488 nm and 560 nm using a confocal microscope after 2 days.

### 4.9. Yeast One-Hybrid (Y1H) and Yeast Two-Hybrid (Y2H) Assays

For the Y1H assay, the full-length CDS of *DcAP2ERF#96* was cloned into the pB42AD vector. Three tandem repeats of the motif sequences (DRE, CRT, GCC) were cloned into the pLacZi2μ vector. The combined plasmids were co-transformed into yeast strain EGY48 and selected on the SD/-Ura/-Trp agar medium. Colonies were then plated onto SD/-Ura/-Trp agar medium containing raffinose, galactose and 5-bromo-4-chloro-3-indolyl-b-D-galactopyranoside (X-gal) for blue color development. For the Y2H assay, the full-length CDS and three truncated fragments of *DcAP2ERF#96* were cloned into the pGBKT7 vector. The constructed plasmids were transformed into AH109 yeast competent with empty pGADT7 using the Yeastmaker Yeast Transformation System (Clontech, Shanghai, China). Transformation cells were plated on SD/-Trp/-Leu medium to screen positive clones. After 4 days, positive clones were transferred to SD/-Ade/-His/-Leu/-Trp dropout medium to detect self-activation. All relevant primer sequences are listed in Table S1.

### 4.10. Acquisition and Handling of Arabidopsis Transgenic Lines

The full-length CDS of *DcAP2ERF#96* was cloned into the pGWB512 vector containing the 35S promoter. *Arabidopsis thaliana* inflorescences were infected by the floral dip method and the selection of transgenic positive lines by MS medium containing hygromycin. The two lines with the highest expression level were identified and selected by qRT-PCR, and their seeds were placed on MS medium for germination to a root length of 1 cm, then transferred to 1.2% MS medium containing 10 μM ABA, and cultured for 5 days before observation phenotype.

## 5. Conclusions

In this study, 120 *AP2/ERF* genes were identified in *D. catenatum* for the first time, and their characteristics were further analyzed. These results contribute to our understanding of the classification and evolution trend of *AP2/ERF* family of orchids. At the same time, according to transcriptome data, we further analyzed the expression pattern of *DcAP2/ERF* family genes and selected 12 genes of interest from them to test their responses to drought stress. In addition, we preliminarily studied the characteristics of transcription factor *DcAP2ERF#96* and found its interacting proteins and promoters according to its inhibition effect on the ABA signaling pathway. However, the specific molecular mechanism of *DcAP2ERF#96* in response to abiotic stress in *D. catenatum* remains to be further studied.

## Figures and Tables

**Table 1:** Characteristics of DcAP2/ERF family proteins.

ID	LOC	Rename	Length (aa)	Mw (Da)	PI	Aliphatic Index	GRAVY	Location
XM_020816501.1	LOC110092113	*DcAP2ERF#1*	393	43,675.56	9.27	59.49	−0.678	mitochondria
XM_020816597.2	LOC110092178	*DcAP2ERF#2*	117	13,244.55	5.3	61.88	−0.913	nucleus
XM_020817122.2	LOC110092532	*DcAP2ERF#3*	205	22,653.27	7.92	67.61	−0.666	nucleus
XM_020817685.2	LOC110092963	*DcAP2ERF#4*	344	38,910.42	4.89	62.21	−0.691	nucleus
XM_020817704.2	LOC110092982	*DcAP2ERF#5*	199	21,704.64	9.36	71.16	−0.545	nucleus
XM_020817821.2	LOC110093058	*DcAP2ERF#6*	142	15,996.03	5.31	67.39	−0.497	mitochondria
XM_020818820.2	LOC110093822	*DcAP2ERF#7*	273	30,323.27	5.12	77.95	−0.467	cytoplasm
XM_020818851.2	LOC110093837	*DcAP2ERF#8*	273	30,499.49	5.28	71.5	−0.49	cytoplasm
XM_020818859.2	LOC110093843	*DcAP2ERF#9*	240	26,225.9	9.69	75.71	−0.383	nucleus
XM_020818985.2	LOC110093926	*DcAP2ERF#10*	378	40,998.34	6.34	56.9	−0.635	nucleus
XM_020819386.2	LOC110094210	*DcAP2ERF#11*	431	47,967.91	6.35	62.48	−0.686	nucleus
XM_020820818.2	LOC110095317	*DcAP2ERF#12*	487	52,737.33	6.81	59.16	−0.591	nucleus
XM_020820987.1	LOC110095445	*DcAP2ERF#13*	258	28,269.68	7.78	66.67	−0.537	nucleus
XM_020821323.2	LOC110095684	*DcAP2ERF#14*	155	17,183.33	9.98	65.03	−0.815	nucleus
XM_020821563.2	LOC110095855	*DcAP2ERF#15*	134	14,806.86	9.71	64.18	−0.525	cytoplasm
XM_020821729.2	LOC110095987	*DcAP2ERF#16*	285	31,434.6	5.44	81.47	−0.432	nucleus
XM_020822089.2	LOC110096240	*DcAP2ERF#17*	219	24,140.1	9.14	59.77	−0.591	cytoplasm
XM_020822222.2	LOC110096335	*DcAP2ERF#18*	231	24,602.71	9.2	68.18	−0.349	nucleus
XM_020822428.2	LOC110096456	*DcAP2ERF#19*	275	29,678.79	6.46	60.51	−0.476	nucleus
XM_020823171.2	LOC110096991	*DcAP2ERF#20*	147	16,174.86	5	64.35	−0.612	nucleus
XM_020823245.2	LOC110097036	*DcAP2ERF#21*	210	22,952.7	8.41	67.57	−0.503	nucleus
XM_020823401.2	LOC110097153	*DcAP2ERF#22*	478	53,136.39	6.52	66.95	−0.597	nucleus
XM_020823475.2	LOC110097214	*DcAP2ERF#23*	181	20,350.63	5.36	61.16	−0.646	nucleus
XM_020823706.2	LOC110097366	*DcAP2ERF#24*	166	18,041.33	6.43	60.6	−0.581	cytoplasm
XM_020824867.2	LOC110098141	*DcAP2ERF#25*	201	21,854.61	5.83	69.1	−0.4	nucleus
XM_020825001.2	LOC110098242	*DcAP2ERF#26*	347	39,167.06	5.64	56.57	−0.847	nucleus
XM_020825318.2	LOC110098477	*DcAP2ERF#27*	323	35,258.22	8.32	58.08	−0.645	nucleus
XM_020825410.2	LOC110098547	*DcAP2ERF#28*	193	20,380.73	4.49	70	−0.454	nucleus
XM_020825656.2	LOC110098714	*DcAP2ERF#29*	174	19,198.48	9.82	58.51	−0.936	nucleus
XM_020825700.2	LOC110098779	*DcAP2ERF#30*	215	23,420.82	6.44	56.88	−0.686	nucleus
XM_020826383.2	LOC110099287	*DcAP2ERF#31*	233	26,407.91	9.26	66.95	−0.771	nucleus
XM_020826509.2	LOC110099386	*DcAP2ERF#32*	239	26,222.62	9.21	74.44	−0.535	nucleus
XM_020826512.1	LOC110099389	*DcAP2ERF#33*	253	28,355.92	6.35	75.89	−0.687	nucleus
XM_020826786.2	LOC110099586	*DcAP2ERF#34*	255	28,810.6	6.22	65.73	−0.436	cytoplasm
XM_020827298.2	LOC110099956	*DcAP2ERF#35*	170	19,093.62	5.69	86.06	−0.573	cytoplasm
XM_020827868.2	LOC110100389	*DcAP2ERF#36*	229	24,589.95	8.91	76.77	−0.172	nucleus
XM_020827870.1	LOC110100391	*DcAP2ERF#37*	164	18,123.61	4.86	86.34	−0.033	cytoplasm
XM_020827871.2	LOC110100392	*DcAP2ERF#38*	164	18,035.27	4.59	85.67	−0.157	cytoplasm
XM_020827872.1	LOC110100393	*DcAP2ERF#39*	178	19,751.31	4.35	83.99	−0.19	cytoplasm
XM_020827873.1	LOC110100394	*DcAP2ERF#40*	122	13,445.6	9.27	96.89	0.025	cytoplasm
XM_020827909.2	LOC110100414	*DcAP2ERF#41*	179	19,288.48	4.51	69.44	−0.491	nucleus
XM_020828052.2	LOC110100522	*DcAP2ERF#42*	165	18,544.94	8.11	65.88	−0.66	cytoplasm
XM_020828120.2	LOC110100557	*DcAP2ERF#43*	268	30,011.64	5.2	67.8	−0.532	nucleus
XM_020828122.2	LOC110100558	*DcAP2ERF#44*	289	31,823.9	5.4	74.05	−0.407	cytoplasm
XM_020828709.2	LOC110100983	*DcAP2ERF#45*	155	16,800.78	4.83	69.48	−0.427	nucleus
XM_020828734.2	LOC110100999	*DcAP2ERF#46*	210	23,031.59	6.44	63.76	−0.604	nucleus
XM_020829170.2	LOC110101314	*DcAP2ERF#47*	733	81,463.46	6.82	64.6	−0.577	nucleus
XM_020829570.2	LOC110101604	*DcAP2ERF#48*	174	18,520.72	6.82	66.32	−0.37	nucleus
XM_020829885.2	LOC110101821	*DcAP2ERF#49*	244	27,139.94	9.23	79.22	−0.437	nucleus
XM_020830439.2	LOC110102213	*DcAP2ERF#50*	338	36,717.66	9.39	69.85	−0.426	nucleus
XM_020831356.2	LOC110102864	*DcAP2ERF#51*	191	21,091.72	9.12	60.94	−0.654	nucleus
XM_020831553.2	LOC110103007	*DcAP2ERF#52*	333	36,879.23	5.83	71.02	−0.514	nucleus
XM_020831585.2	LOC110103039	*DcAP2ERF#53*	205	22,548.56	6.92	63.85	−0.415	nucleus
XM_020831635.2	LOC110103078	*DcAP2ERF#54*	350	39,719.18	8.63	62.74	−0.763	nucleus
XM_020831820.2	LOC110103210	*DcAP2ERF#55*	319	34,146.41	5.23	55.49	−0.573	nucleus
XM_020832156.2	LOC110103436	*DcAP2ERF#56*	233	26,398.05	9.59	61.55	−0.839	nucleus
XM_020832246.1	LOC110103500	*DcAP2ERF#57*	216	23,871.26	4.65	63.7	−0.469	mitochondria
XM_020832253.2	LOC110103508	*DcAP2ERF#58*	188	20,706.09	5.44	55.74	−0.648	nucleus
XM_020832331.2	LOC110103559	*DcAP2ERF#59*	333	37,914.71	6.23	52.82	−0.984	nucleus
XM_020832378.2	LOC110103593	*DcAP2ERF#60*	182	19,679.18	5.35	84.95	−0.175	nucleus
XM_020834156.2	LOC110104876	*DcAP2ERF#61*	226	24,995.95	5.17	76.42	−0.376	nucleus
XM_020834164.2	LOC110104880	*DcAP2ERF#62*	138	15,094.93	5.44	76.45	−0.46	cytoplasm
XM_020834254.2	LOC110104943	*DcAP2ERF#63*	317	35,188.21	5.56	66.81	−0.568	nucleus
XM_020834863.1	LOC110105385	*DcAP2ERF#64*	146	16,196.67	10.1	66.99	−0.646	nucleus
XM_020835088.2	LOC110105539	*DcAP2ERF#65*	351	39,673.69	9.75	58.38	−0.915	nucleus
XM_020835287.2	LOC110105686	*DcAP2ERF#66*	237	26,183.23	7.06	69.58	−0.675	nucleus
XM_020835818.2	LOC110106077	*DcAP2ERF#67*	388	42,370.13	9.25	57.24	−0.649	nucleus
XM_020836334.2	LOC110106428	*DcAP2ERF#68*	193	21,736.5	6.19	66.27	−0.59	cytoplasm
XM_020836335.2	LOC110106429	*DcAP2ERF#69*	259	28,586.49	8.96	81.85	−0.388	nucleus
XM_020837398.2	LOC110107204	*DcAP2ERF#70*	628	69,816.29	6.17	59.54	−0.671	nucleus
XM_020837735.2	LOC110107464	*DcAP2ERF#71*	388	41,489.16	7.81	55.67	−0.557	nucleus
XM_020838142.2	LOC110107770	*DcAP2ERF#72*	266	28,625.14	8.89	73.46	−0.421	nucleus
XM_020838192.2	LOC110107803	*DcAP2ERF#73*	251	27,642.36	5.67	71.95	−0.498	nucleus
XM_020838224.2	LOC110107825	*DcAP2ERF#74*	188	20,344.91	9.3	54.68	−0.677	cytoplasm
XM_020838231.2	LOC110107829	*DcAP2ERF#75*	481	53,471.97	7.64	71.81	−0.516	nucleus
XM_020838234.2	LOC110107830	*DcAP2ERF#76*	476	52,887.25	7.64	71.13	−0.535	nucleus
XM_020838388.2	LOC110107938	*DcAP2ERF#77*	295	32,730.65	5.57	67.83	−0.523	nucleus
XM_020838721.2	LOC110108187	*DcAP2ERF#78*	377	42,334.33	5	60.34	−0.746	nucleus
XM_020838765.2	LOC110108219	*DcAP2ERF#79*	376	41,253.26	6.28	59.26	−0.623	nucleus
XM_020838815.2	LOC110108252	*DcAP2ERF#80*	205	21,740.44	9.34	67.27	−0.423	nucleus
XM_020838948.2	LOC110108340	*DcAP2ERF#81*	250	26,683.77	8.56	64.16	−0.449	nucleus
XM_020839309.2	LOC110108607	*DcAP2ERF#82*	177	19,603.15	7.81	59.77	−0.459	cytoplasm
XM_020839427.2	LOC110108677	*DcAP2ERF#83*	381	41,657.98	5.13	58.71	−0.519	nucleus
XM_020840002.2	LOC110109092	*DcAP2ERF#84*	143	15,701.74	9.51	62.94	−0.524	nucleus
XM_020840101.2	LOC110109163	*DcAP2ERF#85*	146	16,192.68	10.1	66.99	−0.652	nucleus
XM_020840120.2	LOC110109181	*DcAP2ERF#86*	256	26,949.36	9.2	69.1	−0.32	nucleus
XM_020840182.2	LOC110109222	*DcAP2ERF#87*	372	40,701.75	4.68	59.87	−0.761	nucleus
XM_020840524.2	LOC110109455	*DcAP2ERF#88*	263	28,948.72	5.12	48.37	−0.681	nucleus
XM_020840628.2	LOC110109529	*DcAP2ERF#89*	323	35,656.47	4.85	62.29	−0.542	nucleus
XM_020840999.2	LOC110109796	*DcAP2ERF#90*	194	21,956.89	6.31	72.99	−0.591	nucleus
XM_020841375.2	LOC110110072	*DcAP2ERF#91*	326	35,893.82	9.64	78.34	−0.444	nucleus
XM_020841449.2	LOC110110123	*DcAP2ERF#92*	190	21,631.67	8.98	65.32	−0.522	nucleus
XM_020841821.2	LOC110110379	*DcAP2ERF#93*	307	34,245.39	5.85	70.26	−0.536	nucleus
XM_020842046.2	LOC110110526	*DcAP2ERF#94*	377	41,376.99	5.87	62.65	−0.665	nucleus
XM_020842532.1	LOC110110883	*DcAP2ERF#95*	249	27,664.27	9.46	70.92	−0.62	nucleus
XM_020843531.2	LOC110111594	*DcAP2ERF#96*	428	47,796.64	5.64	59.53	−0.581	cytoplasm
XM_020844032.2	LOC110111961	*DcAP2ERF#97*	393	43,606.04	9.3	62.39	−0.647	nucleus
XM_020844150.2	LOC110112064	*DcAP2ERF#98*	213	23,769.37	5	54.23	−0.665	cytoplasm
XM_020844655.2	LOC110112434	*DcAP2ERF#99*	180	19,766.05	6.63	61.28	−0.763	nucleus
XM_020844798.2	LOC110112545	*DcAP2ERF#100*	482	53,271.16	6.47	68.05	−0.595	nucleus
XM_020844875.2	LOC110112594	*DcAP2ERF#101*	311	34,278.27	5.29	70.96	−0.433	nucleus
XM_020844987.2	LOC110112684	*DcAP2ERF#102*	640	69,342.86	6.03	61.78	−0.474	nucleus
XM_020845292.2	LOC110112910	*DcAP2ERF#103*	198	21,803.57	5.71	69.49	−0.397	nucleus
XM_020845879.2	LOC110113332	*DcAP2ERF#104*	160	17,799.22	9.78	62.19	−0.819	nucleus
XM_020846166.2	LOC110113533	*DcAP2ERF#105*	271	30,721.33	5.26	56.24	−0.851	nucleus
XM_020846170.2	LOC110113537	*DcAP2ERF#106*	384	42,198.39	4.73	54.24	−0.736	nucleus
XM_020846319.2	LOC110113656	*DcAP2ERF#107*	350	39,815.18	4.89	55.54	−0.903	nucleus
XM_020847119.2	LOC110114282	*DcAP2ERF#108*	198	21,677.03	6.84	59.29	−0.67	nucleus
XM_020847550.2	LOC110114618	*DcAP2ERF#109*	262	28,562.38	6.78	76.45	−0.426	cytoplasm
XM_020847791.2	LOC110114791	*DcAP2ERF#110*	268	29,590.17	5.9	56.16	−0.538	nucleus
XM_020848468.2	LOC110115279	*DcAP2ERF#111*	138	14,972.24	7.86	84.93	−0.132	nucleus
XM_020848863.2	LOC110115597	*DcAP2ERF#112*	271	30,293.53	5.19	57.68	−0.744	nucleus
XM_020849558.2	LOC110116094	*DcAP2ERF#113*	205	21,591.06	7.7	57.85	−0.299	mitochondria
XM_020850163.2	LOC110116551	*DcAP2ERF#114*	251	28,723.56	9.24	67.21	−0.648	cytoplasm
XM_028691742.1	LOC110112950	*DcAP2ERF#115*	279	31,067.05	6.16	69.21	−0.6	nucleus
XM_028692461.1	LOC110096264	*DcAP2ERF#116*	329	36,673.85	5.29	64.62	−0.631	cytoplasm
XM_028694856.1	LOC110099516	*DcAP2ERF#117*	182	20,153.72	9.35	64.45	−0.713	nucleus
XM_028696782.1	LOC110098115	*DcAP2ERF#118*	201	21,811.55	5.83	68.11	−0.396	nucleus
XM_028698356.1	LOC110103792	*DcAP2ERF#119*	628	67,877	5.8	60.25	−0.61	nucleus
XM_028699460.1	LOC110115745	*DcAP2ERF#120*	248	26,592.45	9.01	51.57	−0.605	nucleus

## Data Availability

All the other datasets supporting the conclusions of this article are included within the article and its additional files.

## Supplementary Materials

The following supporting information can be downloaded at: https://www.mdpi.com/article/10.3390/ijms232113603/s1.

## References

[1] Gutterson N., Reuber T.L. (2004). Regulation of disease resistance pathways by AP2/ERF transcription factors. Curr. Opin. Plant Biol..

[2] Toledo-Ortiz G., Huq E., Quail P.H. (2003). The Arabidopsis Basic/Helix-Loop-Helix Transcription Factor Family. Plant Cell.

[3] Dubos C., Stracke R., Grotewold E., Weisshaar B., Martin C., Lepiniec L. (2010). MYB transcription factors in Arabidopsis. Trends Plant Sci..

[4] Yang X., Tuskan G.A., Cheng Z.-M. (2006). Divergence of the Dof Gene Families in Poplar, Arabidopsis, and Rice Suggests Multiple Modes of Gene Evolution after Duplication. Plant Physiol..

[5] Networks of WRKY Transcription Factors in Defense Signaling. networks-of-wrky-transcription-factors-in-defense-signaling-5d5wsqb4ip.pdf.

[6] Feng K., Hou X.-L., Xing G.-M., Liu J.-X., Duan A.-Q., Xu Z.-S., Li M.-Y., Zhuang J., Xiong A.-S. (2020). Advances in AP2/ERF super-family transcription factors in plant. Crit. Rev. Biotechnol..

[7] Okamuro J.K., Caster B., Villarroel R., Van Montagu M., Jofuku K.D. (1997). The AP2 domain of *APETALA2* defines a large new family of DNA binding proteins in *Arabidopsis*. Proc. Natl. Acad. Sci. USA.

[8] Ohme-Takagi M., Shinshi H. (1995). Ethylene-lnducible DNA Binding Proteins That lnteract with an Ethylene-Responsive Element. Plant Cell.

[9] Allen M.D., Yamasaki K., Ohme-Takagi M., Tateno M., Suzuki M. (1998). A novel mode of DNA recognition by a β-sheet revealed by the solution structure of the GCC-box binding domain in complex with DNA. EMBO J..

[10] Riechmann J.L., Meyerowitz E. (1998). The AP2/EREBP Family of Plant Transcription Factors. Biol Chem..

[11] Nakano T., Suzuki K., Fujimura T., Shinshi H. (2006). Genome-Wide Analysis of the ERF Gene Family in Arabidopsis and Rice. Plant Physiol..

[12] François L., Verdenaud M., Fu X., Ruleman D., Dubois A., Vandenbussche M., Bendahmane A., Raymond O., Just J., Bendahmane M. (2018). A miR172 target-deficient AP2-like gene correlates with the double flower phenotype in roses. Sci. Rep..

[13] Matías-Hernández L., Aguilar-Jaramillo A.E., Marín-González E., Suárez-López P., Pelaz S. (2014). RAV genes: Regulation of floral induction and beyond. Ann. Bot..

[14] Liu Q., Kasuga M., Sakuma Y., Abe H., Miura S., Yamaguchi-Shinozaki K., Shinozaki K. (1998). Two Transcription Factors, DREB1 and DREB2, with an EREBP/AP2 DNA Binding Domain Separate Two Cellular Signal Transduction Pathways in Drought- and Low- Temperature-Responsive Gene Expression, Respectively, in Arabidopsis. Plant Cell.

[15] Zhang T., Cui Z., Li Y., Kang Y., Song X., Wang J., Zhou Y. (2021). Genome-Wide Identification and Expression Analysis of MYB Transcription Factor Superfamily in Dendrobium catenatum. Front. Genet..

[16] Xing H., Jiang Y., Zou Y., Long X., Wu X., Ren Y., Li Y., Li H.-L. (2021). Genome-wide investigation of the AP2/ERF gene family in ginger: Evolution and expression profiling during development and abiotic stresses. BMC Plant Biol..

[17] Riaz M.W., Lu J., Shah L., Yang L., Chen C., Mei X.D., Xue L., Manzoor M.A., Abdullah M., Rehman S. (2021). Expansion and Molecular Characterization of AP2/ERF Gene Family in Wheat (*Triticum aestivum* L.). Front. Genet..

[18] Chen J., Zhou Y., Zhang Q., Liu Q., Li L., Sun C., Wang K., Wang Y., Zhao M., Li H. (2020). Structural variation, functional differentiation and expression characteristics of the AP2/ERF gene family and its response to cold stress and methyl jasmonate in Panax ginseng C.A. Meyer. PLoS ONE.

[19] Ghorbani R., Zakipour Z., Alemzadeh A., Razi H. (2020). Genome-wide analysis of AP2/ERF transcription factors family in Brassica napus. Physiol. Mol. Biol. Plants.

[20] Stockinger E.J., Gilmour S.J., Thomashow M.F. (1997). *Arabidopsis thaliana CBF1* encodes an AP2 domain-containing transcriptional activator that binds to the C-repeat/DRE, a cis-acting DNA regulatory element that stimulates transcription in response to low temperature and water deficit. Proc. Natl. Acad. Sci. USA.

[21] Gilmour S.J., Zarka D.G., Stockinger E.J., Salazar M.P., Houghton J.M., Thomashow M.F. (1998). Low temperature regulation of theArabidopsisCBF family of AP2 transcriptional activators as an early step in cold-inducedCORgene expression. Plant J..

[22] Qin F., Kakimoto M., Sakuma Y., Maruyama K., Osakabe Y., Tran L.-S.P., Shinozaki K., Yamaguchi-Shinozaki K. (2007). Regulation and functional analysis of ZmDREB2A in response to drought and heat stresses in Zea mays L: ZmDREB2A in drought and heat stress response. Plant J..

[23] Zhang G., Chen M., Chen X., Xu Z., Li L., Guo J., Ma Y. (2010). Isolation and characterization of a novel EAR-motif-containing gene GmERF4 from soybean (*Glycine max* L.). Mol. Biol. Rep..

[24] Lee D.-K., Jung H., Jang G., Jeong J.S., Kim Y.S., Ha S.-H., Do Choi Y., Kim J.-K. (2016). Overexpression of the *OsERF71* Transcription Factor Alters Rice Root Structure and Drought Resistance. Plant Physiol..

[25] Zheng K., Cai Y., Chen W., Gao Y., Jin J., Wang H., Feng S., Lu J. (2021). Development, Identification, and Application of a Germplasm Specific SCAR Marker for Dendrobium officinale Kimura et Migo. Front. Plant Sci..

[26] Wan X., Zou L.-H., Zheng B.-Q., Tian Y.-Q., Wang Y. (2018). Transcriptomic profiling for prolonged drought in Dendrobium catenatum. Sci. Data.

[27] Zeng X., Ling H., Chen X., Guo S. (2019). Genome-wide identification, phylogeny and function analysis of GRAS gene family in Dendrobium catenatum (Orchidaceae). Gene.

[28] Zhang G.-Q., Xu Q., Bian C., Tsai W.-C., Yeh C.-M., Liu K.-W., Yoshida K., Zhang L.-S., Chang S.-B., Chen F. (2016). The Dendrobium catenatum Lindl. genome sequence provides insights into polysaccharide synthase, floral development and adaptive evolution. Sci. Rep..

[29] Zhang G.-Q., Liu K.-W., Li Z., Lohaus R., Hsiao Y.-Y., Niu S.-C., Wang J.-Y., Lin Y.-C., Xu Q., Chen L.-J. (2017). The Apostasia genome and the evolution of orchids. Nature.

[30] Niu Z., Zhu F., Fan Y., Li C., Zhang B., Zhu S., Hou Z., Wang M., Yang J., Xue Q. (2021). The chromosome-level reference genome assembly for Dendrobium officinale and its utility of functional genomics research and molecular breeding study. Acta Pharm. Sin. B.

[31] Shrestha A., Fendel A., Nguyen T.H., Adebabay A., Kullik A.S., Benndorf J., Leon J., Naz A.A. (2022). Natural diversity uncovers *P5CS1* regulation and its role in drought stress tolerance and yield sustainability in barley. Plant Cell Environ..

[32] Kasuga M., Liu Q., Miura S., Yamaguchi-Shinozaki K., Shinozaki K. (1999). Improving plant drought, salt, and freezing tolerance by gene transfer of a single stress-inducible transcription factor. Nat. Biotechnol..

[33] Du L., Huang X., Ding L., Wang Z., Tang D., Chen B., Ao L., Liu Y., Kang Z., Mao H. (2022). TaERF87 and TaAKS1 synergistically regulate TaP5CS1/TaP5CR1—mediated proline biosynthesis to enhance drought tolerance in wheat. New Phytol..

[34] Sheng L., Ma C., Chen Y., Gao H., Wang J. (2021). Genome-Wide Screening of AP2 Transcription Factors Involving in Fruit Color and Aroma Regulation of Cultivated Strawberry. Genes.

[35] Wessler S.R. (2005). Homing into the origin of the AP2 DNA binding domain. Trends Plant Sci..

[36] Goyal N., Bhatia G., Sharma S., Garewal N., Upadhyay A., Upadhyay S.K., Singh K. (2020). Genome-wide characterization revealed role of NBS-LRR genes during powdery mildew infection in Vitis vinifera. Genomics.

[37] Sakuma Y., Maruyama K., Osakabe Y., Qin F., Seki M., Shinozaki K., Yamaguchi-Shinozaki K. (2006). Functional Analysis of an *Arabidopsis* Transcription Factor, DREB2A, Involved in Drought-Responsive Gene Expression. Plant Cell.

[38] Roy S.W., Penny D. (2007). A Very High Fraction of Unique Intron Positions in the Intron-Rich Diatom Thalassiosira pseudonana Indicates Widespread Intron Gain. Mol. Biol. Evol..

[39] Magnani E., Sjölander K., Hake S. (2004). From Endonucleases to Transcription Factors: Evolution of the AP2 DNA Binding Domain in Plants. Plant Cell.

[40] Hernandez-Garcia C.M., Finer J.J. (2014). Identification and validation of promoters and cis-acting regulatory elements. Plant Sci..

[41] Zou C., Sun K., Mackaluso J.D., Seddon A.E., Jin R., Thomashow M.F., Shiu S.-H. (2011). *Cis*-regulatory code of stress-responsive transcription in *Arabidopsis thaliana*. Proc. Natl. Acad. Sci. USA.

[42] Agurla S., Gahir S., Munemasa S., Murata Y., Raghavendra A.S., Iwaya-Inoue M., Sakurai M., Uemura M. (2018). Mechanism of Stomatal Closure in Plants Exposed to Drought and Cold Stress. Survival Strategies in Extreme Cold and Desiccation.

[43] Nakashima K., Yamaguchi-Shinozaki K. (2013). ABA signaling in stress-response and seed development. Plant Cell Rep..

[44] Ahn H., Jung I., Shin S.-J., Park J., Rhee S., Kim J.-K., Jung W., Kwon H.-B., Kim S. (2017). Transcriptional Network Analysis Reveals Drought Resistance Mechanisms of AP2/ERF Transgenic Rice. Front. Plant Sci..

[45] Zhao Q., Hu R., Liu D., Liu X., Wang J., Xiang X., Li Y. (2020). The AP2 transcription factor *NtERF172* confers drought resistance by modifying *NtCAT*. Plant Biotechnol. J..

[46] Park C.S., Go Y.S., Suh M.C. (2016). Cuticular wax biosynthesis is positively regulated by WRINKLED4, an AP2/ERF-type transcription factor, in Arabidopsis stems. Plant J..

[47] Letunic I., Bork P. (2018). 20 years of the SMART protein domain annotation resource. Nucleic Acids Res..

[48] Jin J., Tian F., Yang D.-C., Meng Y.-Q., Kong L., Luo J., Gao G. (2017). PlantTFDB 4.0: Toward a central hub for transcription factors and regulatory interactions in plants. Nucleic Acids Res..

[49] Kumar S., Stecher G., Tamura K. (2016). MEGA7: Molecular Evolutionary Genetics Analysis Version 7.0 for Bigger Datasets. Mol. Biol. Evol..

[50] Lu S., Wang J., Chitsaz F., Derbyshire M.K., Geer R.C., Gonzales N.R., Gwadz M., Hurwitz D.I., Marchler G.H., Song J.S. (2020). CDD/SPARCLE: The conserved domain database in 2020. Nucleic Acids Res..

[51] Bailey T.L., Johnson J., Grant C.E., Noble W.S. (2015). The MEME Suite. Nucleic Acids Res..

[52] Lescot M. (2002). PlantCARE, a database of plant cis-acting regulatory elements and a portal to tools for in silico analysis of promoter sequences. Nucleic Acids Res..

[53] Wu Z.-G., Jiang W., Chen S.-L., Mantri N., Tao Z.-M., Jiang C.-X. (2016). Insights from the Cold Transcriptome and Metabolome of Dendrobium officinale: Global Reprogramming of Metabolic and Gene Regulation Networks during Cold Acclimation. Front. Plant Sci..

[54] Li C., Shen Q., Cai X., Lai D., Wu L., Han Z., Zhao T., Chen D., Si J. (2021). JA signal-mediated immunity of Dendrobium catenatum to necrotrophic Southern Blight pathogen. BMC Plant Biol..

[55] Chai D., Lee S.M., Ng J.H., Yu H. (2007). l-Methionine sulfoximine as a novel selection agent for genetic transformation of orchids. J. Biotechnol..

