# Supplementary material for: Genome-Wide Identification of AP2/ERF Transcription Factor Family and Functional Analysis of DcAP2/ERF#96 Associated with Abiotic Stress in Dendrobium catenatum
Source: Int J Mol Sci. 2022 Nov 6;23(21):13603. doi: 10.3390/ijms232113603 (PMC9659080; doi:10.3390/ijms232113603)
Supplement: Supplementary file 1 [file ijms-23-13603-s001.zip › Supplementary Figures S1-S6 Talbes S1-S2.pdf]

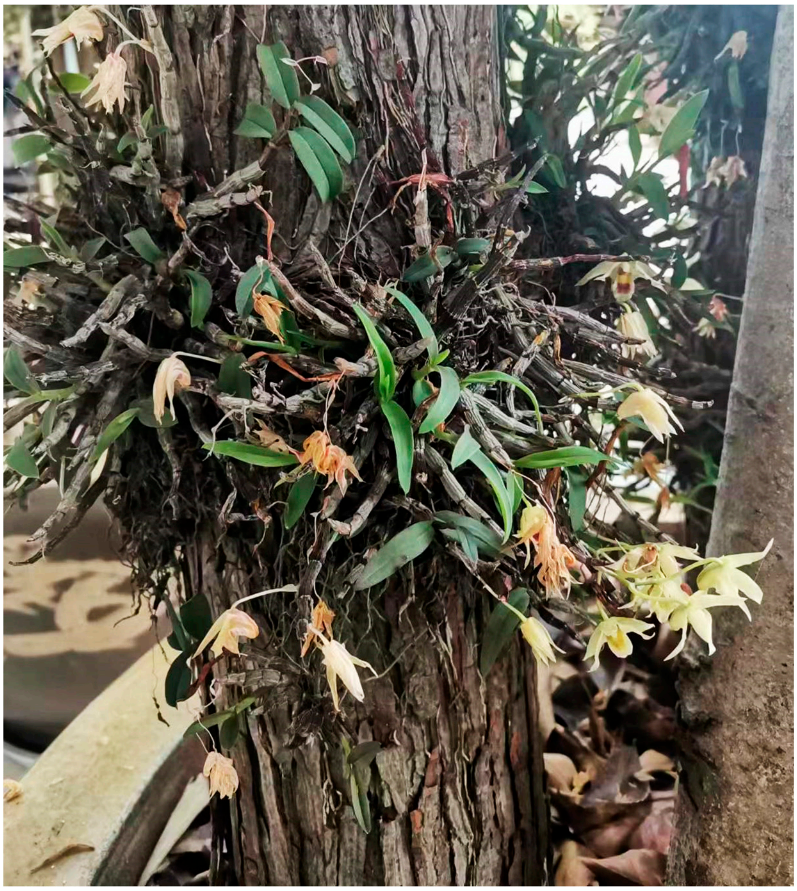

Figure S1. ***Dendrobium catenatum*** **growing on the bark.**

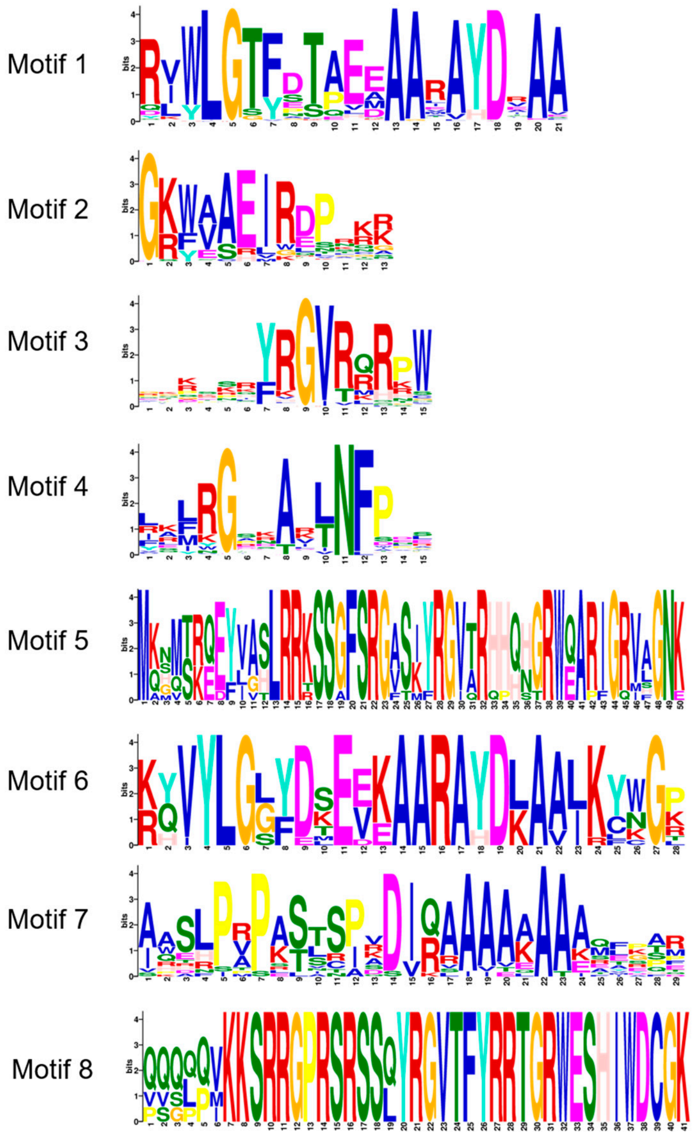

Figure S2. Eight motif logos of DcAP2/ERF family proteins of *Dendrobium catenatum*. The frequency of amino acids is represented by the height of letters.

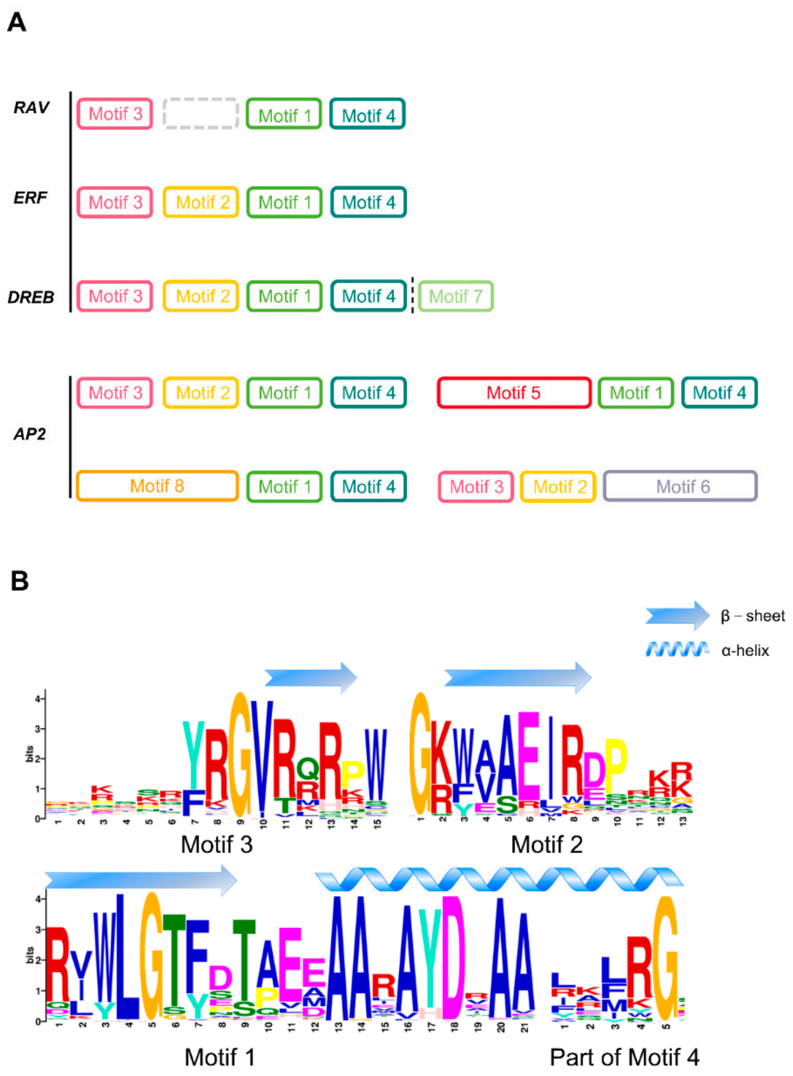

Figure S3. *DcAP2/ERF* family domain analysis.A. Basic motif composition of AP2 domains of each *DcAP2/ERF* subfamily protein. Different motifs are represented by different colors. **B.** Motif composition of typical AP2 domain of *DcAP2/ERF* family protein. The frequency of amino acid occurrences at each position is indicated by letter size.

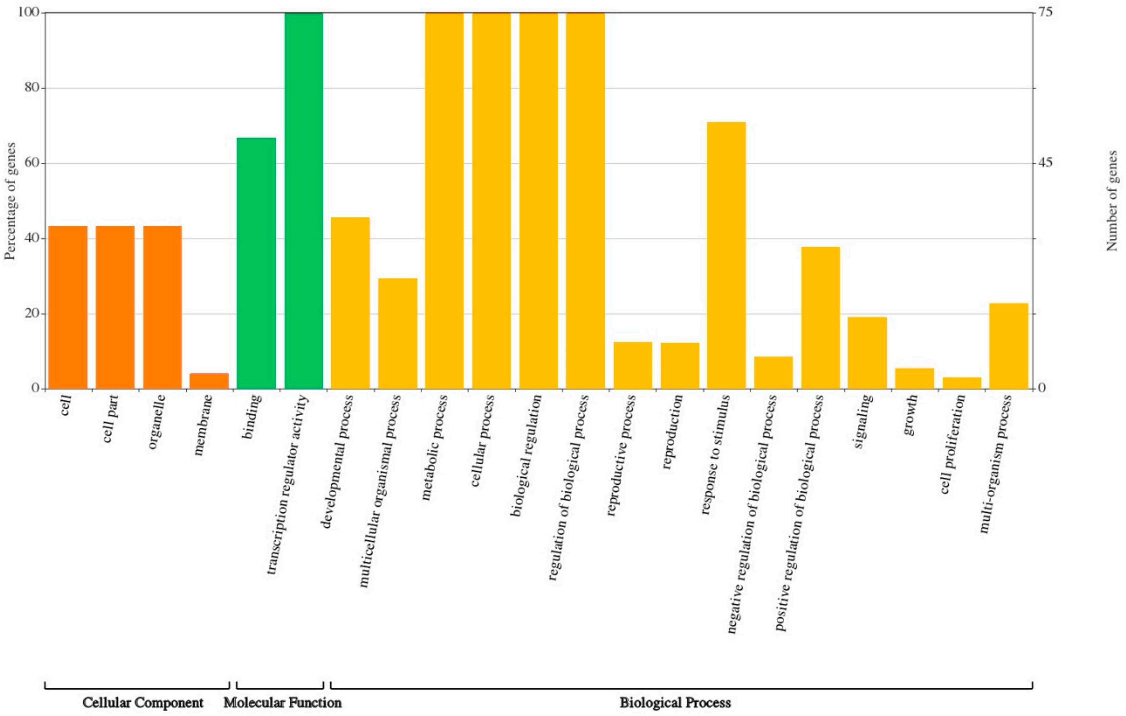

Figure S4. Gene ontology (GO) annotation. The GO terms were showed on the X axis, the percentage of genes and the number of genes were showed on the left and right Y axis respectively.

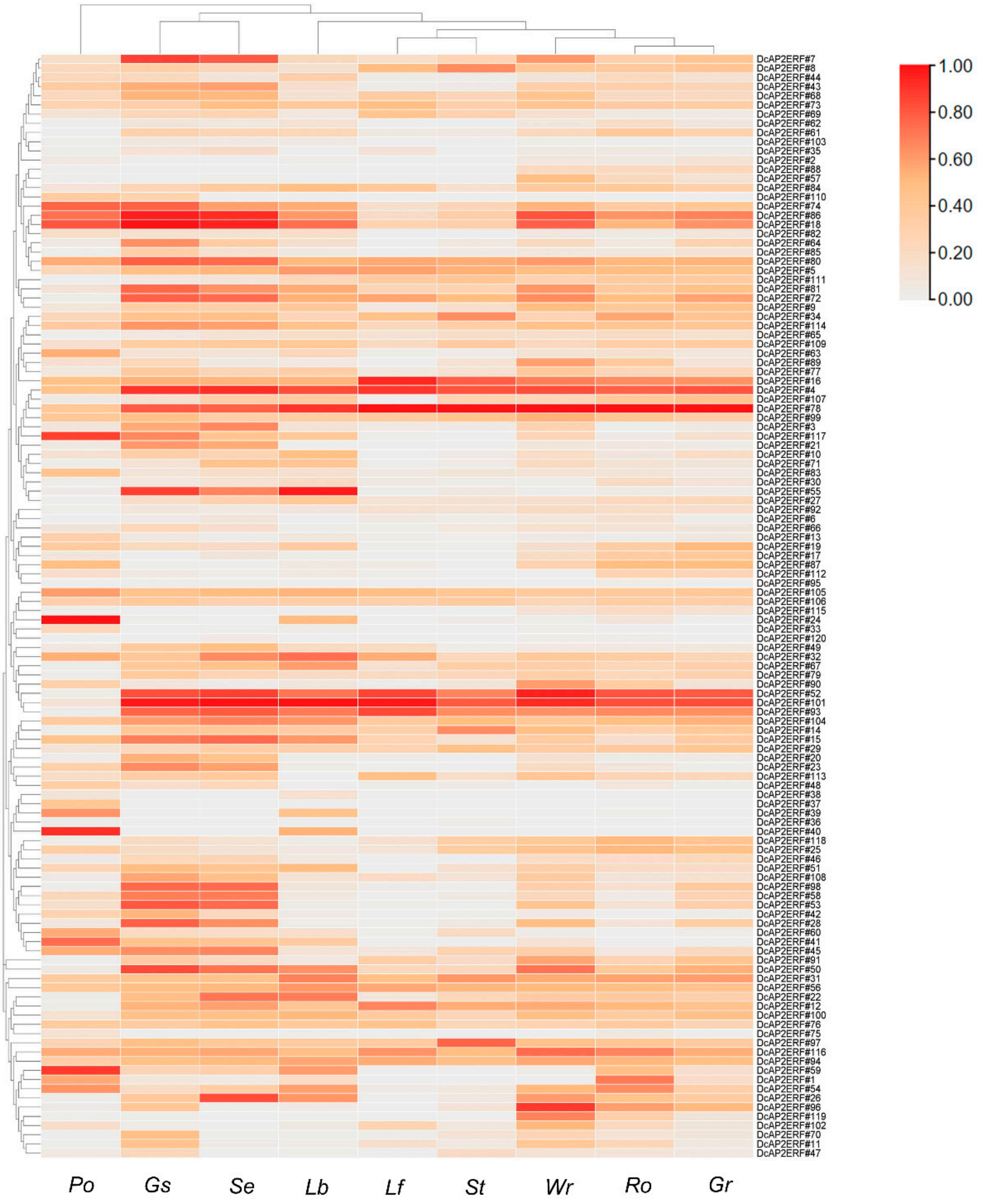

Figure S5. Expression profiles of *DcAP2/ERF* genes in different tissues and organs. The depth of red indicates the level of gene expression. Po: pollinia, Gs, gynostemium, Se: sepal, Lb: labellum, Lf: leaf, St: stem, Wr: white part of root, Ro: root, Gr: green root tip.

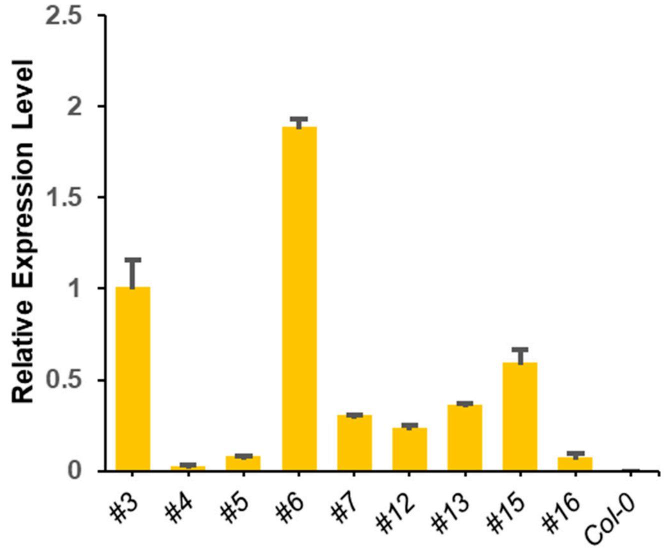

Figure S6. Expression analysis of *DcAP2ERF#96-OE* transgenic lines.
AtACTIN2 was used as an internal control. Values are presented as means ± SD (n = 3).

Table S1. Primer sequences used for research.

Primer name	Sequence (5' to3')
Dcactin-qF	GAGGCACAATCAAAGCGTGG
Dcactin-qR	CAAAGAGAGCACTGCCTGGA
DcAP2ERF#1-qF	TCGTGCTTATGACCTGGCTG
DcAP2ERF#1-qR	TGGACTGGCCTTGCATTTCT
DcAP2ERF#12-qF	TTTCGACACCGAAGTGGAGG
DcAP2ERF#12-qR	GTGCTCAGCTTGAACAGTCG
DcAP2ERF#16-qF	GCGGTTTATCCGCCACTTTC
DcAP2ERF#16-qR	CAGACATCGCCGTAGGACTC
DcAP2ERF#50-qF	GCGGAGGACTCATGTGTTGA
DcAP2ERF#50-qR	CAAGAAGCTCCACAGGGTGA
DcAP2ERF#52-qF	AAAGGCAGGGGATACCGTTG
DcAP2ERF#52-qR	GCCTCCATCGCTTCTAGACC
DcAP2ERF#54-qF	ATTGAAGCTTTTCCGCGGTG
DcAP2ERF#54-qR	CGTTCTGCCGGAGATTAGGG
DcAP2ERF#56-qF	ATGGGAAGCTCGGATTGGTC
DcAP2ERF#56-qR	ATGCCGCGGTATTTGATTGC
DcAP2ERF#78-qF	GACTAGTCGCCGATCACTGG
DcAP2ERF#78-qR	TTCACCAGAGGCGGAAGAAC
DcAP2ERF#86-qF	AATTGGTCTGGAGTGAGGCG
DcAP2ERF#86-qR	TCGCTACCAGTTGCCATCAG
DcAP2ERF#93-qF	GAAGCTGTGTCCATTGTGCC
DcAP2ERF#93-qR	AATGGTTTCGGTGATGGCCT
DcAP2ERF#96-qF	GTTAGCGCCGGCAAAAGAAA
DcAP2ERF#96-qR	GTCGTAGACCTTGGCAGCTT
DcAP2ERF#104-qF	ATATTGGGGCGCTGGAACTC
DcAP2ERF#104-qR	CCCATCTGGTGCTTTGAGGT
AtADH1_qF	ATGGTGGGGTGGACAGGA
AtADH1_qR	AACACCCCAGCCATCGTG
AtABI5_qF	AGAGGTGGTGGTAGCGGT
AtABI5_qR	CTAGCCGCAGTCTCACCG
AtP5CS1_qF	CGCCAAGTGGATAGCGCT
AtP5CS1_qR	CCCCACCTCTGCACCAAG
AtABF4_qF	GAAGGTAGCAGCGGAGGC
AtABF4_qR	AACCCCAGCACGGAACAG
AtABI1_qF	TCGGTGTTTACGACGGCC
AtABI1_qR	CTCCTCCGCCAAAGCCAA
AtABF3_qF	CCAAAGAGCGCCCTGGAT
AtABF3_qR	GCGGGATCTTGCAGCTGA
AtRD22_qF	GGACTTGGTTCGCGGGAA
AtRD22_qR	GGCACCGTTTCAGCCTCT
AtRD29A_qF	CGGAATCTGACGGCGGTT

AtRD29A_qR	CCGTCGGCACATCCTTGT
AtRAB18_qF	CAGGTGGCCAAGGGTACG
AtRAB18_qR	TGGAGTTGCTCTTGGCCG
Atactin2-qF	CTCTCCTTGATCGCCAGTGGTC
Atactin2-qR	TAAGGTCACGTCCAGCAAGGTC
DcAP2/ERF#96-GFP-F	GGACTGCGGGTCTCACACCATGGATATGGAGTACTCCTCT
DcAP2/ERF#96-GFP-R	TTCTATCCCAAATATAGGACTTTG
DcAP2/ERF#96-pB42AD-F	TGCCTCTCCCGAATTCATGGATATGGAGTACTCCTCT
DcAP2/ERF#96-pB42AD-R	CGAGTCGGCCGAATTCTTCTATCCCAAATATAGGACTTTG
GCC-F	ATCTGTCGACCTCGAGAGCCGCCAGCCGCCAGCCGCC
GCC-R	GAGCACATGCCTCGAGGGCGGCTGGCGGCTGGCGGCT
DRE-F	ATCTGTCGACCTCGAGACCGACACCGACACCGAC
DRE-R	GAGCACATGCCTCGAGGTCGGTGTCGGTGTCGGT
CRT-F	ATCTGTCGACCTCGAGGCCGACGCCGACGCCGAC
CRT-R	GAGCACATGCCTCGAGGTCGGCGTCGGCGTCGGC
DcAP2/ERF#96-BD-C-F	CATGGAGGCCGAATTCATGGATATGGAGTACTCCTCT
DcAP2/ERF#96-BD-C-R	GCAGGTCGACGGATCCTCATGGAAGTCCGGTGGCCGA
DcAP2/ERF#96-BD-M-F	CATGGAGGCCGAATTCATGATTTACCGCGGCGTCACC
DcAP2/ERF#96-BD-M-R	GCAGGTCGACGGATCCTCATTATGTTGTAGCGGCTCA
DcAP2/ERF#96-BD-N-F	CATGGAGGCCGAATTCATGAGCACCATAGCCAACTGC
DcAP2/ERF#96-BD-N-R	GCAGGTCGACGGATCCTCATTCTATCCCAAATATAGG
DcAP2/ERF#96-BD-F	CATGGAGGCCGAATTCATGGATATGGAGTACTCCTCT
DcAP2/ERF#96-BD-R	GCAGGTCGACGGATCCTCATTCTATCCCAAATATAGG
RAB18-lac-F	ATCTGTCGACCTCGAGACCCAGCCAAGTTTCTTCAA
RAB18-lac-R	GAGCACATGCCTCGAGGTTCTTCTTGTCTTAAGCAA
P5CS1-lac-F	ATCTGTCGACCTCGAGGAAATCGTGAGAGACGACGT
P5CS1-lac-R	GAGCACATGCCTCGAGTATCGTCGTCGTCGTCTACC
RD29A-lac-F	ATCTGTCGACCTCGAGGTTCTTGACATCATTCAATT
RD29A-lac-R	GAGCACATGCCTCGAGTCCAATAGAAGTAATCAAAC
DREB2A-1-AD-F	GGAGGCCAGTGAATTCATGGCGGAAGAAGAAAGGAA
DREB2A-1-AD-R	CGAGCTCGATGGATCCTGGTAAATCTGAAAAGAAGC
DREB2A-2-AD-F	GGAGGCCAGTGAATTCATGGCGGAGCCCGAAAGGAA
DREB2A-2-AD-R	CGAGCTCGATGGATCCGAAAAGGCCACCTCCATCAG

Table S2. GO annotation of DcAP2/ERF gene family.

Gene ID	GO ID	Gene ID	GO ID
DcAP2ERF#98	GO:0001101	DcAP2ERF#41	GO:0001067
DcAP2ERF#96	GO:0003002	DcAP2ERF#40	GO:0001067
DcAP2ERF#95	GO:0000302	DcAP2ERF#4	GO:0001666
DcAP2ERF#93	GO:0000160	DcAP2ERF#39	GO:0001067
DcAP2ERF#91	GO:0000003	DcAP2ERF#38	GO:0001067
DcAP2ERF#90	GO:0003674	DcAP2ERF#37	GO:0001067
DcAP2ERF#89	GO:0000003	DcAP2ERF#36	GO:0001067
DcAP2ERF#88	GO:0000003	DcAP2ERF#35	GO:0000160
DcAP2ERF#87	GO:0000302	DcAP2ERF#33	GO:0001067
DcAP2ERF#85	GO:0000160	DcAP2ERF#32	GO:0003674
DcAP2ERF#80	GO:0000160	DcAP2ERF#30	GO:0003674
DcAP2ERF#8	GO:0001067	DcAP2ERF#29	GO:0001101
DcAP2ERF#78	GO:0001666	DcAP2ERF#28	GO:0001067
DcAP2ERF#77	GO:0000003	DcAP2ERF#27	GO:0003674
DcAP2ERF#73	GO:0001067	DcAP2ERF#26	GO:0003674
DcAP2ERF#70	GO:0003674	DcAP2ERF#25	GO:0003674
DcAP2ERF#7	GO:0001067	DcAP2ERF#24	GO:0001067
DcAP2ERF#69	GO:0001067	DcAP2ERF#22	GO:0000003
DcAP2ERF#68	GO:0001067	DcAP2ERF#21	GO:0003674
DcAP2ERF#66	GO:0000160	DcAP2ERF#20	GO:0001101
DcAP2ERF#64	GO:0000160	DcAP2ERF#17	GO:0000302
DcAP2ERF#63	GO:0000003	DcAP2ERF#16	GO:0003674
DcAP2ERF#62	GO:0000160	DcAP2ERF#15	GO:0001101
DcAP2ERF#61	GO:0000160	DcAP2ERF#14	GO:0001101
DcAP2ERF#60	GO:0001067	DcAP2ERF#120	GO:0000003
DcAP2ERF#59	GO:0000981	DcAP2ERF#12	GO:0000003
DcAP2ERF#58	GO:0001101	DcAP2ERF#118	GO:0003674
DcAP2ERF#52	GO:0000160	DcAP2ERF#115	GO:0001067
DcAP2ERF#50	GO:0000003	DcAP2ERF#112	GO:0000302
DcAP2ERF#5	GO:0000160	DcAP2ERF#111	GO:0003674
DcAP2ERF#49	GO:0003674	DcAP2ERF#11	GO:0003674
DcAP2ERF#47	GO:0003674	DcAP2ERF#107	GO:0001666
DcAP2ERF#46	GO:0003674	DcAP2ERF#106	GO:0001067
DcAP2ERF#45	GO:0001067	DcAP2ERF#105	GO:0001067
DcAP2ERF#44	GO:0001067	DcAP2ERF#104	GO:0001101
DcAP2ERF#43	GO:0001067	DcAP2ERF#103	GO:0000160
DcAP2ERF#42	GO:0003674	DcAP2ERF#101	GO:0000160
DcAP2ERF#1	GO:0000981		

Table S3. DcAP2/ERF Family Promoter Details.
(In separate Excel table "Table S3")

Table S4. Differential expression analysis of *DcAP2/ERF* family under low temperature treatment.
(In separate Excel table "Table S4")

Table S5. Differential expression analysis of *DcAP2/ERF* family under MeJA treatment.
(In separate Excel table "Table S5")
